# Postsynaptic Targeting and Mobility of Membrane Surface-Localized hASIC1a

**DOI:** 10.1007/s12264-020-00581-9

**Published:** 2020-09-30

**Authors:** Xing-Lei Song, Di-Shi Liu, Min Qiang, Qian Li, Ming-Gang Liu, Wei-Guang Li, Xin Qi, Nan-Jie Xu, Guang Yang, Michael Xi Zhu, Tian-Le Xu

**Affiliations:** 1 Center for Brain Science of Shanghai Children’s Medical Center, Shanghai Jiao Tong University School of Medicine, Shanghai, 200127 China; 2 Department of Anatomy and Physiology, Shanghai Jiao Tong University School of Medicine, Shanghai, 200025 China; 3 Shanghai Research Center for Brain Science and Brain-Inspired Intelligence, Shanghai, 201210 China; 4 Shanghai Institute for Advanced Immunochemical Studies, ShanghaiTech University, Shanghai, 201210 China; 5 Department of Integrative Biology and Pharmacology, McGovern Medical School, University of Texas Health Science Center at Houston, Houston, TX 77030 USA

**Keywords:** ASIC1a, Surface labeling, Visualization, Membrane trafficking, Brain-derived neurotrophic factor, Synaptic function

## Abstract

Acid-sensing ion channels (ASICs), the main H^+^ receptors in the central nervous system, sense extracellular pH fluctuations and mediate cation influx. ASIC1a, the major subunit responsible for acid-activated current, is widely expressed in brain neurons, where it plays pivotal roles in diverse functions including synaptic transmission and plasticity. However, the underlying molecular mechanisms for these functions remain mysterious. Using extracellular epitope tagging and a novel antibody recognizing the hASIC1a ectodomain, we examined the membrane targeting and dynamic trafficking of hASIC1a in cultured cortical neurons. Surface hASIC1a was distributed throughout somata and dendrites, clustered in spine heads, and co-localized with postsynaptic markers. By extracellular pHluorin tagging and fluorescence recovery after photobleaching, we detected movement of hASIC1a in synaptic spine heads. Single-particle tracking along with use of the anti-hASIC1a ectodomain antibody revealed long-distance migration and local movement of surface hASIC1a puncta on dendrites. Importantly, enhancing synaptic activity with brain-derived neurotrophic factor accelerated the trafficking and lateral mobility of hASIC1a. With this newly-developed toolbox, our data demonstrate the synaptic location and high dynamics of functionally-relevant hASIC1a on the surface of excitatory synapses, supporting its involvement in synaptic functions.

**Electronic supplementary material:**

The online version of this article (10.1007/s12264-020-00581-9) contains supplementary material, which is available to authorized users.

## Introduction

Fluctuations in extracellular pH occur commonly in the brain during many physiological and pathological processes [1–5]. As major proton sensors, acid-sensing ion channels (ASICs) play several distinct roles in the central and peripheral nervous systems [6–9]. ASICs belong to the degenerin and epithelial Na^+^ channel (DEG/ENaC) superfamily [10]. In rodents, there are three genes encoding five isoforms: ASIC1a, 1b, 2a, 2b, and 3, which can form homo- and heterotrimeric channels in various combinations, with different pH sensitivity, ion selectivity, as well as activation and desensitization kinetics [11–13]. The closely-related isoforms ASIC4 and brain-liver-intestine amiloride-sensitive Na^+^ channel (BLINaC), are unable to produce or modify H^+^-evoked currents [14, 15]. Although ASIC1a, 2a, and 2b are all widely expressed in the brain [16–18], ASIC1a is the key determinant of acid-evoked current in neurons [19–21], while ASIC2 plays important modulatory roles [22]. Typically, the activation of ASICs induces neuronal depolarization accompanied by Ca^2+^ entry into the cell through either direct or indirect mechanisms [23, 24].

ASIC1a has been implicated in many brain functions, such as synaptic plasticity [21, 25–27], fear and anxiety [28–31], and neuronal injury [20, 32–35]. While the molecular and cellular mechanisms of these regulatory actions are important areas to explore, the subcellular localization of the channels in specialized neuronal compartments also serves to define its physiological roles. Endogenous ASIC1a is abundant in synaptosomes prepared by biochemical fractionation using density gradient centrifugation [21]. While the antibody against the C-terminus of ASIC1a has revealed its presence in the dendrites, but not axons, of dissociated mouse cortical and hippocampal neurons [21, 23], the antibody against the extracellular domain of ASIC1a showed a universal distribution in cultured central nervous system neurons [36]. These discrepant observations, on one hand, provide clues about the subcellular location of ASIC1a in neurons, but on the other hand, they underscore the difficulty of studying the endogenous channels. ASIC1a appears to be preferentially localized in dendrites, especially at excitatory synapses, as evidenced by immunohistochemistry in cultured hippocampal slices and primary neurons transfected with N-terminus-tagged ASIC1a [21, 23]. However, it is unclear if the spine expression actually reflects the localization of the functional channels on synaptic membranes. The cell-surface distribution of ASIC1a in neurons, especially with respect to extra-synaptic and synaptic regions, also remains undefined.

Adding to the complexity is the difference between rodent and human ASIC1a. Although the mouse (m) and human (h) ASIC1a proteins share 98% amino-acid identity, studies have shown that hASIC1a generates a larger acid-activated current than mASIC1a in heterologous expression systems [37]. This may be attributed to a single amino-acid substitution at position 285, which increases the N-glycosylation and cell surface expression of hASIC1a, and as a result, acidotoxicity mediated by hASIC1a appears to be more severe than that by mASIC1a [38]. Indeed, resected cortical tissue from humans exhibit a higher membrane/total ratio of ASIC1a than that from mice [38]. Electrophysiological recordings of acutely cultured cortical neurons also revealed pH-dependent differences in activation and inactivation as well as the rate of recovery from inactivation between native hASIC1a and mASIC1a [39]. In addition, the overexpression of hASIC1a, but not mASIC1a, in rat organotypic cultures of hippocampal slices results in increased spine density [23, 37]. These findings suggest that hASIC1a exhibits a greater response to acid signaling and has a stronger impact on the related biological effects than mASIC1a. More in-depth investigations of hASIC1a are, therefore, necessary in order to gain better insights into the role of hASICs in brain function and disease.

In the present study, we applied new tools, extracellular epitope-tagged hASIC1a and an hASIC1a ectodomain antibody to examine its cell-surface distribution and trafficking in cortical neurons. We showed surface-exposed hASIC1a puncta to exhibit rapid movement in excitatory synapses and dendritic segments, which is increased by brain-derived neurotrophic factor (BDNF), a key modulator of neuronal development and differentiation [40–45] and neural plasticity [46–54]. The findings and new tools introduced here will enable a better understanding of the contributions of hASIC1a to synaptic function and learning and memory, as well as acidosis-related pathological conditions.

## Materials and Methods

### Culture of Primary Rodent Cortical Neurons and Cell Line

Animal care and the experimental protocols were approved by the Animal Ethics Committee of Shanghai Jiao Tong University School of Medicine, Shanghai, China (permit number: DLAS-MP-ANIM.01-05). The conventional global ASIC1a-knockout (*Asic1a*^−/−^) mice were the generous gifts of Professor Michael J. Welsh (Howard Hughes Medical Institute, University of Iowa, Iowa City, IA, USA). Dissociated neurons were prepared and maintained as previously described [55, 56]. Briefly, cerebral cortices from 15–18 day embryonic Sprague Dawley rats or *Asic1a*^−/−^ mice were dissected in D-Hank’s solution and dissociated by 0.05% trypsin for 15 min. Cells were plated (~2×10^5^ cells/35-mm dish for electrophysiology and immunocytochemistry; ~2×10^6^ cells/60-mm dish for biochemistry) on poly-D-lysine coated cover glasses or dishes. Cultures were maintained in Neurobasal medium containing 2% B27 and 1% Glutamax supplements at 37°C in a 5% CO_2_ humidified atmosphere.

Chinese hamster ovary (CHO) K1 cells were grown in F12K medium supplemented with 10% fetal bovine serum (FBS), 1% penicillin/streptomycin, and 1% Glutamax supplements at 37°C in a 5% CO_2_ humidified atmosphere.

### Reagents and Antibodies

All drugs and reagents were from Sigma unless otherwise indicated. The main reagents were as follows: Neurobasal medium (Gibco), B27 Supplement (Gibco), Sulfo-NHS-LC-Biotin (Thermo Scientific), NeutrAvidin Agarose Resins (Thermo Scientific), recombinant human BDNF (R&D System), and K252a (Tocris).

The antibodies used for western blotting were ASIC1a (1:500, sc-13905, Santa Cruz) and GAPDH (1:2000, KC-5G4, KangChen). The HRP-conjugated secondary antibodies used for western blotting were goat anti-rabbit IgG (1:2000, AP132P, Millipore), rabbit anti-mouse IgG (1:2000, AP160P, Millipore), and rabbit anti-goat IgG (1:2000, AP106P, Millipore). The primary antibodies used for immunostaining were GFP (1:1000, A10262, Invitrogen), HA (1:1000, 901501, BioLegend), DsRed (1:1000, 632496, Clontech), and PSD-95 (1:1000, ab13552, Abcam). The secondary antibodies used for immunostaining were donkey anti-human IgG DyLight 550 (1:1000, SA5-10127, ThermoFisher), donkey anti-human IgG DyLight 488 (1:1000, SA5-10126, ThermoFisher), donkey anti-mouse IgG Alexa 647 (1:1000, 715-605-150, Jackson ImmunoResearch), donkey anti-rabbit IgG Alexa 568 (1:1000, A10042, Invitrogen), and goat anti-chicken IgG Alexa 488 (1:1000, A11039, Invitrogen).

### Plasmids and Transfection

cDNA of human ASIC1a was cloned into the pEGFP-C3 vector (Clontech) or the pcDNA3 vector. For the GFP-hASIC1a plasmid, GFP was linked at the N-terminus of human ASIC1a. For the hASIC1a-HA plasmid, the hemagglutinin (HA) epitope (YPYDVPDYA) of influenza virus was inserted in the extracellular loop of hASIC1a: one or two copies between residues Phe^147^ and Lys^148^ [57, 58], or one copy between Asp^298^ and Leu^299^. For the hASIC1a-^298^pHluorin^299^ plasmid, the HA tag between Asp^298^ and Leu^299^ was replaced by the pH-sensitive GFP variant pHluorin.

CHO-K1 cells were transiently transfected using HilyMax liposome transfection reagent (Dojindo Laboratories). Electrophysiological recordings or immunostaining was performed 16 h–24 h after transfection. Calcium phosphate transfection was performed on cultured neurons at 7 days–10 days *in vitro* (DIV). Before transfection, the original medium was changed to fresh Neurobasal medium. In a 35-mm dish, 1 μg–4 μg of plasmid was mixed with 60 μL CaCl_2_ (0.3 mol/L) by pipetting up and down, then 60 μL 2× HBSS (in mmol/L: 280 NaCl, 1.5 Na_2_HPO_4_, 50 HEPES, pH 6.9) was added. After fully mixing, the transfection solution was immediately transferred into the dish with the neurons. After incubation at 37°C for 1 h–1.5 h, the medium was replaced with CO_2_-saturated Neurobasal medium (wash medium) to remove excess calcium phosphate particles. After that, the wash medium was replaced with 1 mL of the original medium and 1 mL of fresh Neurobasal medium plus B27 supplement and the dish returned to the culture incubator. At 18–21 DIV, immunostaining or live cell imaging experiments were performed.

### Preparation of Antibody Against hASIC1a Ectodomain Based on Screening of Combinatorial Antibody Library

Differential enrichment-based screening of the combinatorial antibody library for hASIC1a antibody preparation was as described previously [59]. Briefly, a truncated hASIC1a with amino-acids 13–464 (ΔhASIC1a) was incorporated into the lipid nanodisc as the antigen. The hASIC1a-specific scFv antibodies were selected from a combinatorial human monoclonal scFv antibody phage library (10^11^ diversity) after three rounds of screening; these were then constructed into full-length IgG1 antibody format. HEK293F cells were transfected for antibody production. The antibody was purified by affinity binding to a Protein A HP column (GE Healthcare). The purified antibodies were then concentrated (15 mg/mL) and stored in phosphate buffered saline (PBS), pH 7.4, at −80°C.

### Surface hASIC1a Labeling, Immunocytochemistry, and Image Acquisition

Surface hASIC1a was stained as described [60] with some modification. Briefly, neurons or CHO cells grown on glass coverslips were incubated with HA antibody or ASC06-IgG1 in culture medium at 37°C for 10 min–15 min. The cells were then washed with pre-warmed standard extracellular solution (ECS, pH 7.4) to remove unbound antibody. The standard ECS contained the following (in mmol/L): 150 NaCl, 5 KCl, 1 MgCl_2_, 2 CaCl_2_, 10 glucose, and 10 HEPES (buffered to a specific pH with TrisBase or HCl). After fixation with 4% paraformaldehyde + 4% sucrose in PBS for 15 min, the samples were blocked in a detergent-free blocking solution (PBS with 5% FBS and 0.02% sodium azide) for 1 h, followed by secondary antibody incubation at room temperature (~22°C) for 1 h. To immunostain other proteins, cultures were then post-fixed in −20°C methanol for 1 min to permeabilize the neurons. The cells were covered with blocking solution for 1 h, followed by incubation with primary antibodies overnight at 4°C. After washing, the cells were incubated with secondary antibody at room temperature for 1 h, and then washed and mounted on glass slides with fluorescent mounting medium (Dako) for imaging. For all immunocytochemical experiments, GFP and mCherry signals were enhanced by staining with primary antibodies for GFP and DsRed, followed by Alexa 488 and 568 conjugated secondary antibodies, respectively. In BDNF experiments, antibody incubation in ECS immediately followed the BDNF/K252a treatment.

All static images were acquired with a Leica SP8 laser scanning confocal microscope with a 63× (NA 1.40) oil-immersion lens. Dendritic regions of interest in cortical neurons were first taken as three-dimensional image stacks and then projected to two-dimensional images using the maximal intensity z-projection function. For z projection, 0.3 μm was taken as the step interval to capture images. The number of planes, typically 5–8, was chosen to cover the entire dendrite. Images for all conditions in a particular experiment were captured using identical acquisition parameters (gain, offset, laser power, pinhole size, and scan speed) and were analyzed with LAS X software (Leica) using identical parameters.

### Surface Protein Biotinylation and Western Blotting

Surface biotinylation was performed on CHO cells and neurons following established protocols [57, 61]. Briefly, cells were washed three times with ice-cold PBS^+/+^ solution (PBS plus 1 mmol/L MgCl_2_ and 0.4 mmol/L CaCl_2_, pH 8.0), followed by the addition of 0.25 mg/ml Sulfo-NHS-LC-Biotin (Thermo Scientific) to the PBS^+/+^ solution at 4°C for 30 min with gentle rocking. Unbound biotin was quenched by a PBS^+/+^ solution containing 0.1 mol/L glycine. Ten percent of the volume of the lysate was saved for determination of total proteins and mixed with 4× Laemmli sample buffer. The remainder was incubated with NeutrAvidin Agarose Resins (Thermo Scientific) overnight at 4°C with gentle agitation for surface protein extraction. Beads were washed three times with PBS containing 1% Triton X-100, and bound proteins were eluted with 2× Laemmli sample buffer. An equal volume of surface and total proteins was loaded per lane and proteins were separated by SDS-PAGE and transferred to PVDF membranes (GE Healthcare). Immunoblotting was performed using the desired primary antibody followed by the corresponding HRP-conjugated secondary antibody and detected by enhanced chemiluminescence (ECL Substrate, Thermo Scientific) using a CCD camera (ImageQuant LAS 4000, GE Healthcare). Quantification was done using ImageJ software.

### Electrophysiology

Axopatch 200B with Digidata 1440A and pClamp10 software (Molecular Devices) were used for whole-cell recordings from CHO cells. Recording pipettes were pulled to 4 MΩ–6 MΩ when filled with the internal solution containing (in mmol/L): 120 KCl, 30 NaCl, 0.5 CaCl_2_, 1 MgCl_2_, 5 EGTA, 2 MgATP, and 10 HEPES, pH 7.2. Cells were recorded at a holding potential of −60 mV. A “Y-tube” system was used to achieve rapid exchange of ECS. ASIC1a channels were activated by a pH drop from 7.4 to pH 6.0 or other values every 2 min to allow complete recovery of the channel from desensitization.

### Fluorescence Recovery After Photobleaching (FRAP)

The FRAP assay was conducted on an A1R laser-scanning confocal microscope (Nikon) equipped with an automated z drive with Perfect Focus, multiple laser lines with acousto-optic tunable filter control, and a motorized x-y stage. Neurons co-transfected with mCherry and hASIC1a-^298^pHluorin^299^, or pHluorin-GluA2, were mounted in a confocal dish containing ECS and imaged before and after bleaching at 20-s intervals using an oil immersion objective (60× 1.40 NA). Photobleaching was done by using a region of interest (ROI) that enclosed a single mushroom spine head or a dendritic segment with a 488-nm laser. In order to minimize the potential influence of residual pHluorin fluorescence after quenching, for image-capture, we conducted photobleaching for 1 full minute with maximal power (100%) of the 488-nm laser on a selected ROI; for data analysis, we calculated the relative pHluorin-GluA2/hASIC1a-pHluorin levels within the ROI at individual time points after subtracting the residual signal. The amount of recovery at each time point was calculated as FRAPt = (*F*_t_ − *F*_0_)/(*F*_i_ − *F*_0_), where F_t_ is the fluorescence in the bleached area at time t, F_0_ is the fluorescence of the same field immediately after bleaching, and F_i_ is the initial intensity of the field before photobleaching. For BDNF treatment, BDNF (20 ng/mL) or BDNF plus K252a (200 nmol/L) was applied to neurons through perfusion for 5 min and the photobleaching was executed during the last minute of the drug treatment. Then the solution was returned to basal ECS for another 20 min.

### Antibody Modifications and Single-Particle Tracking (SPT)

ASC06-IgG1 was buffer-exchanged into PBS using an Amicon Ultra-4 Centrifugal Filter Unit (Millipore) and concentrated to 30 mg/mL prior to modification. Conjugating reagent 2, 5-dioxopyrrolidin-1-yl 4-azidobenzoate (NHS-azide) was dissolved in dimethyl formamide (DMF) in 10 mmol/L stock solution in advance. Direct NHS-azide-antibody conjugates were prepared by reacting antibody and NHS-azide at a 1:10 molar ratio in 0.2 mL PBS containing 10% DMF and 0.1 mol/L NaHCO_3_ (pH 8.5) overnight at room temperature. Excess NHS-azide was removed from the NHS-azide-antibody conjugate by using a Zeba spin desalting column (Thermo Scientific). This was followed by reaction with Alexa Fluor™ 488 DIBO Alkyne (Thermo Scientific) at a 1:10 molar ratio for 4 h at room temperature. NHS-azide-antibody-Alexa 488 complex was further purified in PBS using Amicon Ultra-4 centrifugal filtration systems. Antibody concentration was determined by absorption measurement.

For SPT experiments, *Asic1a*^−/−^ neurons transfected with hASIC1a or untransfected rat cortical neurons were incubated with ASC06-IgG1-Alexa 488 in ECS for 10 min at 37°C. After washing off the free antibodies, single particles were tracked using an oil immersion objective (60× 1.49 NA PlanApoN TIRF) at 20 Hz–30 Hz sampling frequency for 1–2 min in a Delta Vision OMX imaging system (GE). Single-particle trajectories were reconstructed using the Spots mode of the Imaris software (Bitplane AG). An MSD (mean square displacement) *versus* time plot [62–64] was calculated using the MSD Plot function of the Imaris software to quantify the lateral mobility of surface hASIC1a puncta on different dendritic areas or following stimulation. Note that, in order to focus on channels with a single antibody label, a low concentration of the conjugated antibody was used and only particles with fluorescent intensities that fell within the major Gaussian curve (leftmost) in the frequency distribution histogram (Fig. S3) were analyzed. Also, only the trajectories with a tracking time of >500 ms were included in the MSD analysis. For BDNF treatment, the neurons were subjected to BDNF stimulation first and then labeled with ASC06-IgG1-Alexa 488 for SPT.

### Statistical Analysis

Data are presented as the mean ± SEM unless indicated otherwise. Statistical differences were determined by Student’s *t* test for two-group comparisons or by one-way or two-way ANOVA followed by Tukey’s honest significant difference *post hoc* correction for multiple comparisons for more than two groups. Significance was taken as *P* < 0.05 and displayed as *P < 0.05, **P < 0.01, ***P < 0.001, and ****P < 0.0001.

## Results

### Compartmentalization of hASIC1a in Cortical Neurons

We first re-examined the subcellular localization of total hASIC1a in cultured rat primary cortical neurons co-transfected with mCherry and N-terminal GFP-tagged hASIC1a (GFP-hASIC1a) (Fig. S1, A–C). Based on mCherry labeling, we assigned smooth branches with an even diameter as axons and branches with multiple sub-branches and small protrusions (spines) as dendrites (Fig. S1C). We found that, while mCherry was distributed uniformly everywhere, GFP-hASIC1a, as detected using the GFP antibody, mainly co-existed with mCherry in somata and dendrites and was rarely detected in axon-like branches (Fig. S1C). To exclude the potential effect of endogenous ASIC1a on the heterologously-expressed channels, we expressed GFP-hASIC1a in primary cortical neurons prepared from prenatal ASIC1a*-*null mice (*Asic1a*^−/−^) (Fig. S1D). Ratio imaging of immunostained GFP-hASIC1a *versus* mCherry showed enrichment of GFP-hASIC1a in dendritic spines (Fig. S1D). Quantification of the relative intensity revealed the order of GFP-hASIC1a abundance in dendritic spines ≈ somata > dendritic shafts >> axons (Fig. S1E). Pairwise comparisons also showed stronger GFP-hASIC1a signals in the spine head than in the adjacent dendritic shaft region (Fig. S1F and G). To further confirm the preferential dendritic localization of hASIC1a, we co-transfected mCherry-hASIC1a and the axon vesicle marker, GFP-VAMP2, in *Asic1a*^−/−^ neurons and found the VAMP2-positive axons to be mostly free of mCherry-hASIC1a label (Fig. S1H). These results demonstrate the differential compartmentalization of total hASIC1a, which is mainly located in the somata and dendrites of cortical neurons, with preferential presence in dendritic spines, similar to the previous report on the distribution of ASIC1a in hippocampal neurons [23].

### Ex-tagged hASIC1a Constructs for Cell-Surface Labeling

**Fig. 1:**
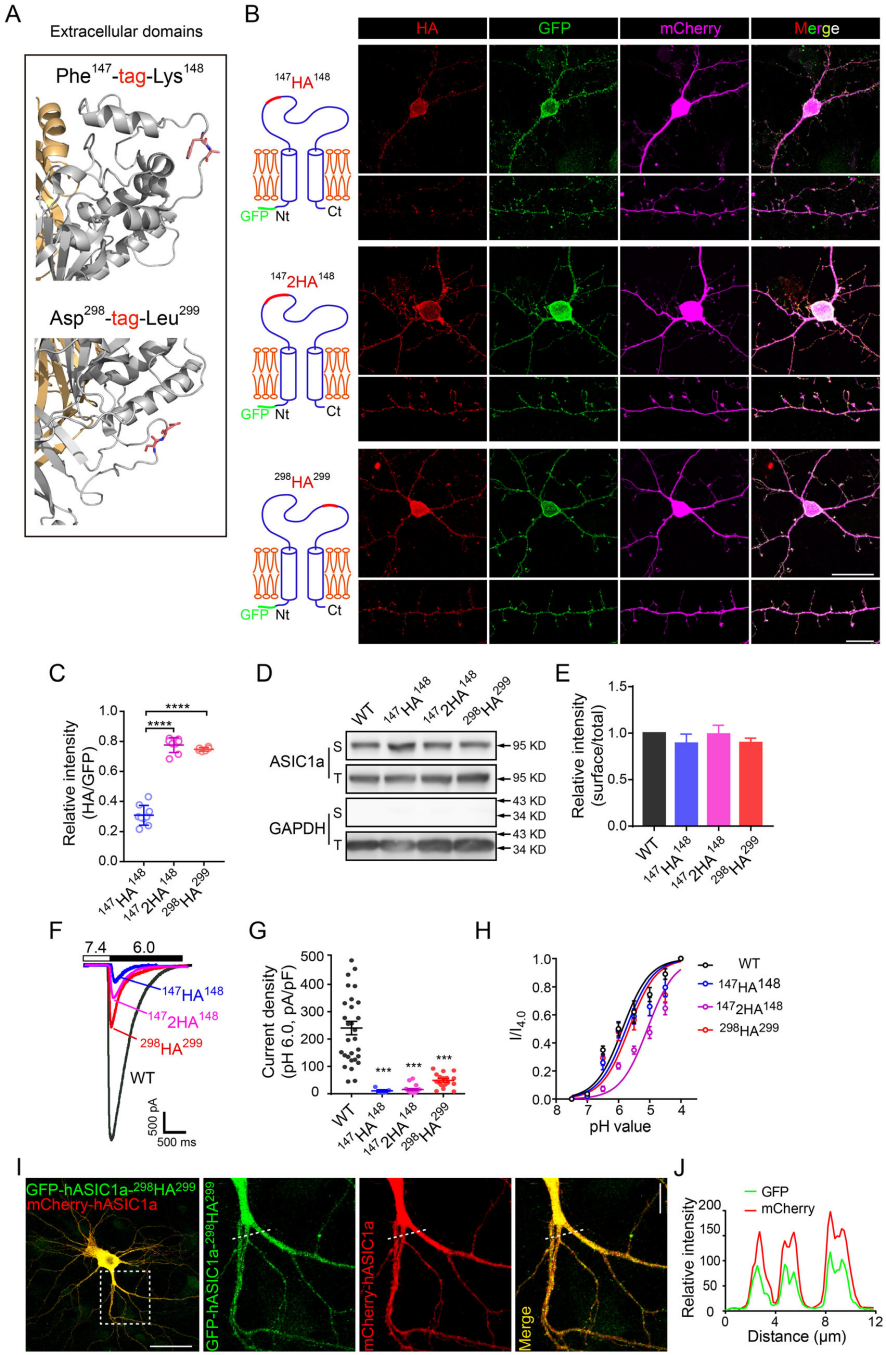
Screening extracellular HA-tagged hASIC1a for surface staining. **A** Candidate sites for epitope tag insertion in the extracellular loop of hASIC1a. Based on the high-resolution structure, two exposed flexible sites were chosen for epitope insertion, the first between Phe^147^ and Lys^148^ (upper panel) and the second between Asp^298^ and Leu^299^ (lower panel). **B** Representative images of surface staining by HA (red) and total hASIC1a expression by GFP (green) in *Asic1a*^−/−^ mouse cortical neurons that expressed different HA-tagged GFP-hASIC1a as indicated at left. mCherry was co-transfected to indicate neuronal morphology (magenta). Scale bars: upper panel, 20 μm; lower panel, 10 μm. **C** Quantification of surface hASIC1a labeling in neurons based on HA/GFP fluorescence intensity ratio. Data are presented as the mean ± SEM. The ratios for ^147^2HA^148^ (0.775 ± 0.018, *n =* 8 cells) and ^298^HA^299^ (0.747 ± 0.005, *n =* 6 cells) are higher than that for ^147^HA^148^ (0.307 ± 0.022, *n =* 9 cells). ****P < 0.0001, no significant difference between ^147^2HA^148^ and ^298^HA^299^, one-way ANOVA multiple comparison. **D**, **E** Representative blots (**D**) and quantification (**E**) of surface (biotinylated, S) and total (T) hASIC1a in CHO cells that expressed WT and the three HA-tagged hASIC1a as indicated. GAPDH was used as a cytoplasmic protein control. The normalized surface/total ratios are presented as the mean ± SEM: ^147^HA^148^, 0.89 ± 0.10; ^147^2HA^148^, 0.99 ± 0.10; ^298^HA^299^, 0.90 ± 0.05. No significant difference between WT and any HA-tagged hASIC1a, one-way ANOVA multiple comparison, *n =* 3 experiments. **F** Representative traces of acid (pH 6.0)-activated currents recorded from CHO cells transfected with WT or HA-tagged hASIC1a as indicated. Cells were held at −60 mV; baseline pH was 7.4. **G** Quantification of peak current density. Data are presented as the mean ± SEM. The density of *I*_6.0_ (pA/pF) for ^147^HA^148^ (10.92 ± 3.80, *n =* 9 cells), ^147^2HA^148^ (15.46 ± 3.57, *n =* 19 cells), and ^298^HA^299^ (48.78 ± 6.54, *n =* 18 cells), is lower than that of WT hASIC1a (259.5 ± 26.61, *n =* 29 cells). ***P < 0.001, one-way ANOVA multiple comparison. **H** pH dose-response curves for WT and HA-tagged hASIC1a (normalized to current elicited by pH 4.0 solution). To ensure that the channels were not desensitized before stimulation, the baseline pH was 9.0. pH_50_ (data are presented as the mean ± SEM): WT, 6.13 ± 0.05 (*n =* 13 cells); ^147^HA^148^, 5.78 ± 0.06 (*n =* 13 cells); ^147^2HA^148^, 5.13 ± 0.04 (*n =* 15 cells); and ^298^HA^299^, 5.67 ± 0.08 (*n =* 12 cells). **I**, **J** GFP-hASIC1a-^298^HA^299^ displays the same localization as WT hASIC1a (represented by mCherry-hASIC1a). GFP-hASIC1a-^298^HA^299^ and mCherry-hASIC1a were co-transfected into *Asic1a*^−/−^ neurons. **I** Representative images of GFP and mCherry, showing excellent co-localization with a Pearson’s coefficient (r) of 0.917. Scale bars: left panel, 50 μm; right panel, 10 μm. **J** Intensity plots for the line profile of GFP and mCherry along the dashed lines shown in (**I**).

ASIC proteins are present both on the plasma membrane (PM) and in intracellular compartments, but only the channels expressed on the PM are functionally relevant for sensing extracellular pH fluctuations. However, it is unclear if the spine expression actually reflects localization of the functional channels on synaptic membranes. To selectively label the channels exposed on the cell surface, an HA epitope was genetically inserted into the extracellular loop of rat ASIC1a (rASIC1a) or hASIC1a between residues Phe^147^ and Lys^148^ (ASIC1a-^147^HA^148^, see Fig. 1A, upper panel). After heterologous expression in *Xenopus* oocytes or CHO cells, the extracellular HA epitope was detected by immunocytochemistry under non-permeabilized conditions to reveal cell-surface ASIC1a [58, 65, 66]. However, when expressed in neurons, these constructs mainly displayed surface labeling in and near the somata [57, 66], with relatively faint staining in dendrites and hardly any signal in spines (Fig. 1B, upper panels).

To overcome this problem, we screened two other constructs based on the high-resolution crystal structures of ASIC1a [67, 68]: one with two joint HA tags inserted between Phe^147^ and Lys^148^ (hASIC1a-^147^2HA^148^, Fig. 1A, upper panel), and another with one HA tag inserted between Asp^298^ and Leu^299^ (hASIC1a-^298^HA^299^, Fig. 1A, lower panel), which are centrally located in two different disordered exposed sites of the extracellular loop. A GFP was fused to the cytoplasmic N-terminal end to aid the detection of total hASIC1a. Upon expression in *Asic1a*^−/−^ neurons, both new constructs revealed surface staining of hASIC1a on somata and dendrites, as well as on dendritic spines (Fig. 1B, middle and lower panels). Quantification revealed higher surface/total ratios for GFP-hASIC1a-^147^2HA^148^ and GFP-hASIC1a-^298^HA^299^ than GFP-hASIC1a-^147^HA^148^ (Fig. 1C). Therefore, the insertion of two HA tags between Phe^147^ and Lys^148^ or just one HA between Asp^298^ and Leu^299^ of hASIC1a improved the exposure of the epitope for antibody recognition under non-permeabilized conditions, allowing better detection of the surface-expressed channels than the original GFP-hASIC1a-^147^HA^148^.

Next, we compared the functionality of these constructs. After expression in CHO cells, acid-activated currents were measured by lowering the extracellular pH from 7.4 to 6.0 (*I*_6.0_) with the cell voltage clamped at −60 mV in the whole-cell configuration. While each of the HA-tagged hASIC1a constructs showed a decrease in *I*_6.0_ compared to the WT channel (GFP-hASIC1a) (Fig. 1F), GFP-hASIC1a-^298^HA^299^ showed the largest *I*_6.0_ and GFP-hASIC1a-^147^HA^148^ had the smallest among the three (Fig. 1F and G). Testing the pH sensitivity of the constructs by holding the cells at pH 9.0 first (to remove all steady-state desensitization) and then switching to the solution to pH values ranging from 7.5 to 4.0, we detected only slight right-shifts in the pH dose-response curves for GFP-hASIC1a-^298^HA^299^ and GFP-hASIC1a-^147^HA^148^ compared to WT hASIC1a (Fig. 1H). However, the dose-response curve of GFP-hASIC1a-^147^2HA^148^ was more dramatically right-shifted (Fig. 1H), indicating a large change in the pH sensitivity of this construct.

To determine whether the HA tag insertion affected the association of hASIC1a with the PM, we biotinylated the surface proteins on the hASIC1a-expressing CHO cells and found that none of the three ex-tagged constructs changed the surface expression as compared to the WT GFP-hASIC1a (Fig. 1D and E). Although biotin only labeled exposed primary amines on the surface in these experiments, the pull-down by streptavidin likely collected proteins and sub-membrane vesicles physically associated with the PM. Therefore, the apparent discrepancy between the results from biotinylation (Fig. 1D and E) and antibody labeling under non-permeabilized conditions (Fig. 1B and C) could suggest normal vesicle trafficking, but varying degrees of impaired insertion into the PM, i.e. a substantial amount of the ex-tagged ASIC1a might be stuck in vesicles right underneath the PM and present in complexes with PM proteins. Moreover, based on the structural dynamics of the hASIC1a extracellular loop, tag insertion in different sites of the hASIC1a ectodomain may affect epitope tag exposure for antibody recognition and channel properties, which may also differentially affect surface intensities and channel gating. Nonetheless, the ex-tagged hASIC1a exhibited a subcellular distribution almost identical to the WT hASIC1a in neurons. In cortical neurons from ASIC1a-null mice co-transfected with GFP-hASIC1a-^298^HA^299^ and WT mCherry-hASIC1a, the fluorescence signals of GFP and mCherry almost perfectly co-localized, with a Pearson’s coefficient (*r*) of 0.917 (Fig. 1I and J).

Collectively, hASIC1a-^298^HA^299^ showed sufficient antibody recognition and surface labeling in neuronal branches, including dendritic spines. The alteration in acid-induced currents was less severe than in the other two HA-tagged constructs, making it suitable for surface hASIC1a labeling. Therefore, we used hASIC1a-^298^HA^299^ for subsequent experiments.

### Distribution of hASIC1a-^298^HA^299^ on Neuronal Plasma Membrane

**Fig. 2:**
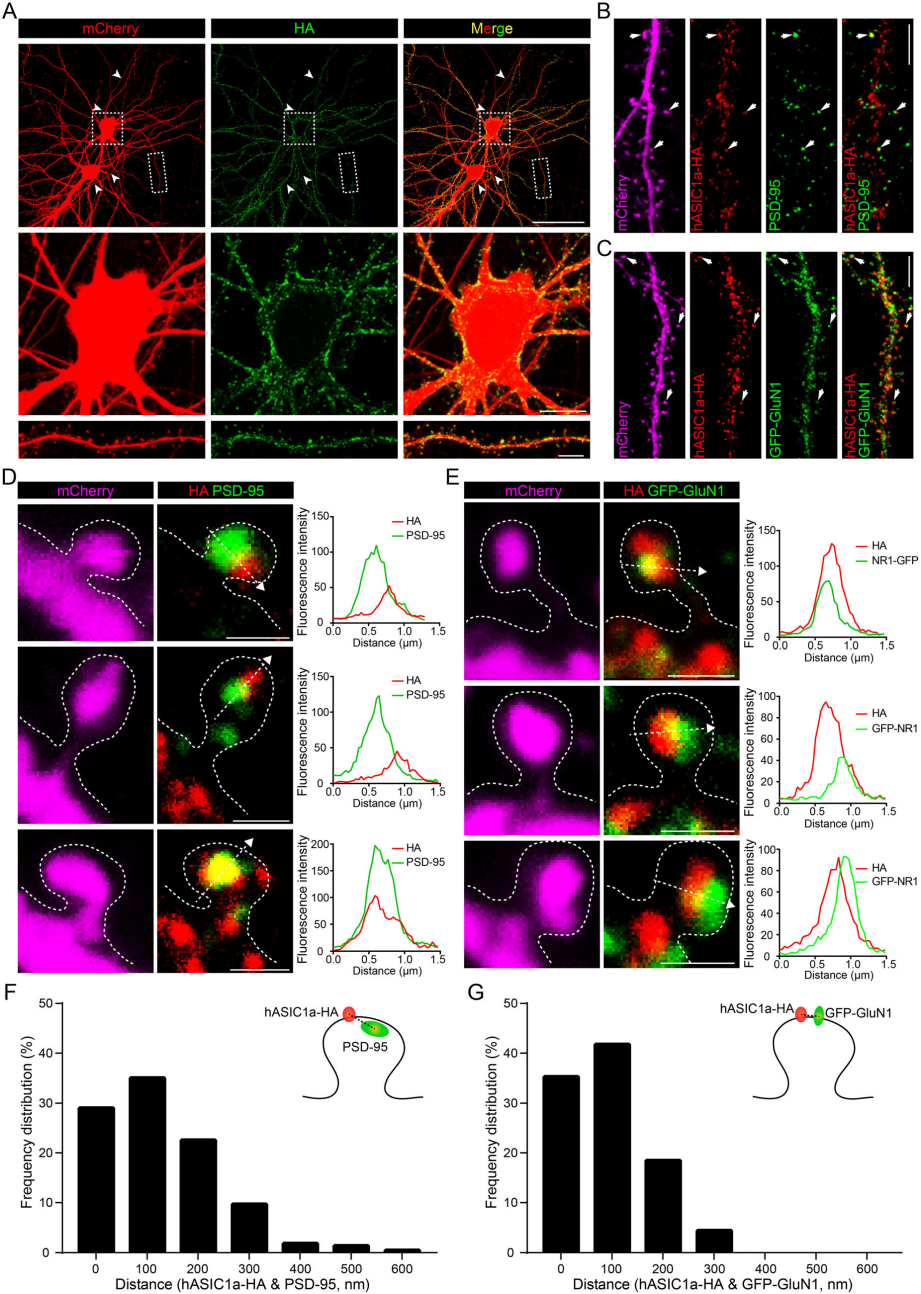
hASIC1a distribution on the plasma membrane of cortical neurons labeled by an extracellular tag. **A** Distribution of neuronal surface hASIC1a labeled by hASIC1a-^298^HA^299^. Surface staining for HA (green) and immunostaining for mCherry (red) in *Asic1a*^−/−^ neurons co-transfected with hASIC1a-^298^HA^299^ and mCherry. Arrowheads indicate axons. Enlarged representative images show surface hASIC1a localization in soma (middle) and a dendrite fragment (lower). Scale bars: upper panel, 50 μm; middle panel, 10 μm; lower panel, 5 μm. **B**, **D** Relative localization of surface hASIC1a with the excitatory synaptic marker PSD-95. **B** Representative images of surface staining for HA (red) and immunostaining for PSD-95 (green) and mCherry (magenta) in *Asic1a*^−/−^ neurons co-transfected with hASIC1a-^298^HA^299^ and mCherry. Scale bar, 5 μm. **D** Representative images of enlarged spines indicated by arrows in (**B**) and line profiling of fluorescence intensity. Plots show the line profiles of HA (red) and PSD-95 (green) along the dashed lines in the labeled spines. Scale bars, 1 μm. **C**, **E** Relative localization of surface hASIC1a with the excitatory synaptic marker GluN1. **C** Representative dendrites with surface staining for HA (red) and immunostaining for GFP-GluN1 (green) and mCherry (magenta) in *Asic1a*^−/−^ neurons co-transfected with hASIC1a-^298^HA^299^, mCherry, and GFP-GluN1. Scale bar, 5 μm. **E** Representative images of enlarged spines indicated by arrows in (**C**) and line profiling of fluorescence intensity. Plots show the line profiles of HA (red) and GFP-GluN1 (green) along the dashed lines in the labeled spines. Scale bars, 1 μm. **F** Localization analysis of surface hASIC1a and PSD-95 at synapses. The distances between the centers of surface hASIC1a cluster and PSD-95 puncta in individual spine heads are characterized by a frequency distribution histogram. Spines with a distance of ≤200 nm account for ~80% (*n =* 217 spines). **G** Localization analysis of surface hASIC1a and GFP-GluN1 at synapses. The distances between the centers of surface hASIC1a clusters and GFP-GluN1 puncta in individual spine heads are characterized by a frequency distribution histogram. Spines with a distance of ≤200 nm account for >90% (*n =* 292 spines).

We carefully examined the dendritic localization of surface channels in *Asic1a*^−/−^ neurons transfected with hASIC1a-^298^HA^299^ and mCherry (Fig. 2A). Under non-permeabilized conditions, the HA labeling was clustered on the PM of somata, dendritic shafts, and spines (Fig. 2A), but not axons (Fig. 2A), consistent with the distribution of total hASIC1a (Fig. S1C and D). These observations demonstrate that functional surface hASIC1a preferentially targets to somatodendritic regions, with clustering on the PM of both dendritic shafts and spines.

Interestingly, by co-immunostaining with the excitatory post-synaptic markers PSD-95 and the NMDA receptor subunit GluN1 (Fig. 2B and C), we found that the surface hASIC1a clusters in spine heads were closely adjacent to or partially co-localized with PSD-95 or GluN1 puncta (Fig. 2D and E). By quantifying the distance between fluorescent signal centers of surface hASIC1a clusters and synaptic marker puncta in mushroom spines, we found that 80%–90% of the dendritic spines displayed very close distance of ≤200 nm between surface hASIC1a and the synaptic markers (Fig. 2F and G). These observations indicate that hASIC1a is present at the surface of excitatory synapses, closely associated with the post-synaptic density region, in line with its role in excitatory synaptic function. Thus, by using hASIC1a-^298^HA^299^, we unveiled the dendritic localization and synaptic targeting of surface hASIC1a in cortical neurons.

### Surface-Exposed Channels in Cortical Neurons Revealed by a Novel hASIC1a Ectodomain Antibody

**Fig. 3:**
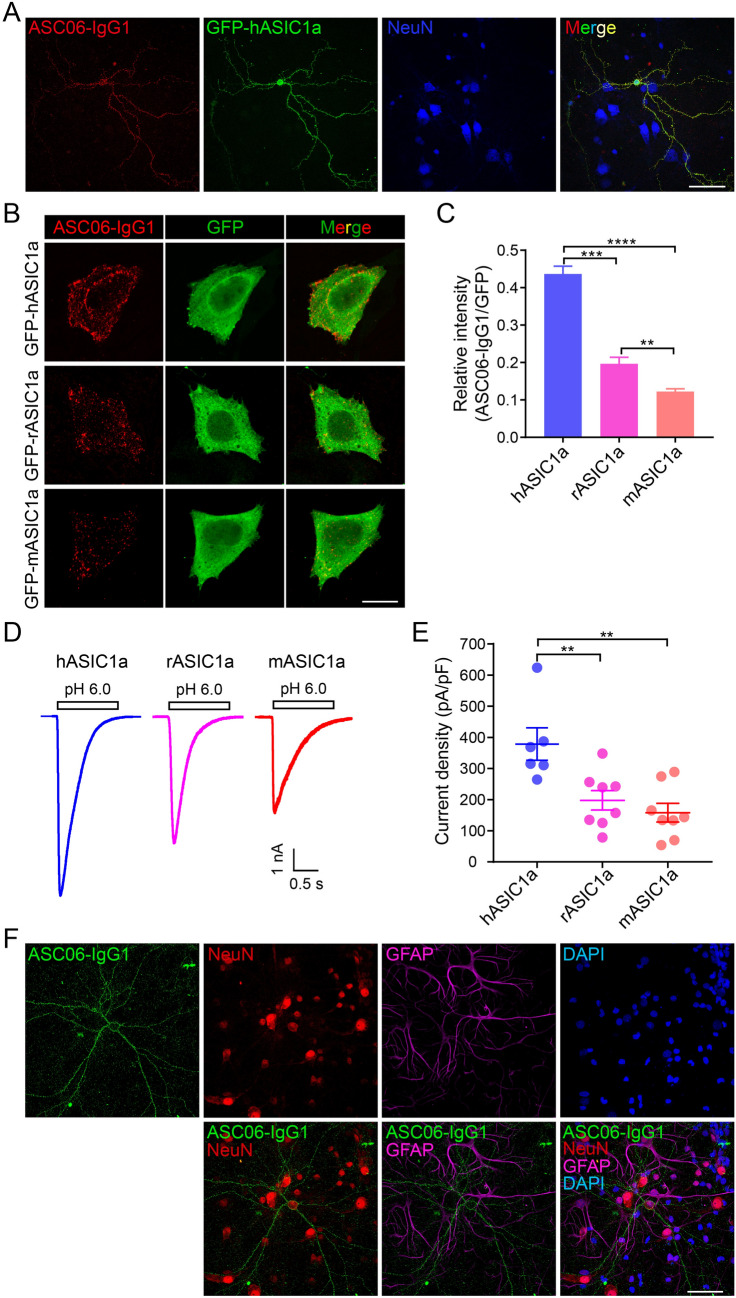
Specificity of the novel antibody against the hASIC1a ectodomain. **A** Verification of antibody specificity in neurons by surface staining with ASC06-IgG1 in *Asic1a*^−/−^ neurons transfected with GFP-hASIC1a. Fluorescent signal of surface hASIC1a occurs only in the transfected neuron. Scale bar, 50 μm. **B**, **C** Species specificity of ASC06-IgG1 against ASIC1a tested by transfecting CHO cells with GFP-tagged hASIC1a, rASIC1a, or mASIC1a and surface staining with ASC06-IgG1. **B** Representative images of surface staining (red) and total ASIC1a expression (green) (scale bar, 20 μm). **C** Quantification of surface labeling based on ASC06-IgG1/GFP fluorescence intensity ratios. Data are presented as the mean ± SEM. [hASIC1a, 0.44 ± 0.02 (*n =* 11 cells); rASIC1a, 0.20 ± 0.02 (*n =* 9 cells); mASIC1a, 0.12 ± 0.01 (*n =* 13 cells); **P < 0.01, ***P < 0.001, ****P < 0.0001, one-way ANOVA multiple comparison]. **D**, **E** Acid-activated current of GFP-hASIC1a, -rASIC1a, or -mASIC1a expressed in CHO cells. Cells were transfected with the same amount of cDNA for the three orthologs followed by whole-cell recording of *I*_6.0_. **D** Representative *I*_6.0_ traces. **E** Quantification of current density of peak *I*_6.0_ (pA/pF). Data are presented as the mean ± SEM. [hASIC1a, 378.47 ± 52.22 (*n =* 6 cells); rASIC1a, 197.73 ± 31.30 (*n =* 8 cells); mASIC1a, 157.98 ± 30.15 (*n =* 8 cells), **P < 0.01, no significant difference between rASIC1a and mASIC1a, one-way ANOVA multiple comparison]. **F** Endogenous rASIC1a detected by ASC06-IgG1 in primary cultures of rat cortical neurons immunostained at DIV 18 with ASC06-IgG1 (green) under non-permeabilized conditions; neurons were then stained with the neuronal marker NeuN (red), astrocyte marker GFAP (magenta), and nuclear maker DAPI (blue) (scale bar, 50 μm).

Although the ex-tagged hASIC1a construct provided the possibility to detect cell-surface hASIC1a, the modified channel did suffer the drawback of lower channel function than WT hASIC1a (Fig. 1F and G). To detect WT ASIC1a on the cell surface, antibodies that recognize the ectodomain of ASIC1a are necessary. Although two ASIC1a antibodies had been used for immunohistochemical examination of the endogenous ASIC1a distribution in neurons, the results are inconsistent as noted earlier [21, 23, 36]. The discrepancy may result from different strategies for antibody preparation and issues with the specificity and sensitivity of the antibodies. Recently, a new antibody, ASC06-IgG1, that recognizes the ectodomain of hASIC1a was developed based on phage-display [59]. To verify the specificity of this antibody, we double-stained for surface hASIC1a and the neuronal marker NeuN in *Asic1a*^−/−^ neurons transfected with GFP-hASIC1a. Only in the transfected neurons with green fluorescence was the surface hASIC1a signal detected (Fig. 3A).

To test whether ASC06-IgG1 could also react with rat and mouse ASIC1a, we transfected GFP-hASIC1a, -rASIC1a, or -mASIC1a into CHO cells. Surface staining showed that the antibody was able to recognize ASIC1a from all three species tested (Fig. 3B), but the intensity of the antibody staining followed the sequence human > rat > mouse, based on the surface/total ratio (Fig. 3C). To determine what causes the difference in labeling efficiency, we recorded acid-activated currents in these cells (Fig. 3D). The peak *I*_6.0_ showed the sequence hASIC1a > rASIC1a > mASIC1a (Fig. 3D and E), consistent with the previous reports demonstrating more efficient membrane trafficking and the resultant larger H^+^-evoked currents of hASIC1a than mASIC1a [37, 38], an effect that had been attributed to Pro^285^ located at the extracellular loop of hASIC1a [38]. These results suggest that more efficient trafficking of hASIC1a to the PM may underlie the better ASC06-IgG1 labeling efficiency of surface-expressed hASIC1a than rodent ASIC1a. However, since the substitutions at amino-acid 285 of rodent channels may also cause conformational changes in the extracellular loop, and quantitatively, the impact of species difference on surface staining was clearly larger than on *I*_6.0_ (compare Fig. 3C and 3E), other factors beyond membrane trafficking cannot be excluded at this point. Given that hASIC1a was used in the original phage display screening that identified the ASC06-IgG1 antibody, it is possible that hASIC1a may confer the conformation better than other orthologs for recognition by this antibody, especially because the epitope recognized by ASC06-IgG1 most likely relies on the 3-D assembly of the hASIC1a trimers rather than the linear sequence [59].

Despite the relatively weaker recognition, we were able to detect surface-expressed endogenous rASIC1a in cultured rat cortical neurons with ASC06-IgG1 and found it to be distributed along the membrane of somata and dendrites (Fig. 3F), similar to the heterologously-expressed hASIC1a-HA (Fig. 2A). Interestingly, the antibody only labeled a small population of the rat neurons, as indicated by co-stained NeuN, while no fluorescent signal of surface rASIC1a was detected in astrocytes labeled with GFAP (Fig. 3F), indicating that ASIC1a is specifically expressed on the surface of at least a subpopulation of rat cortical neurons, but not that of astrocytes.

### Synaptic Localization of Surface hASIC1a Revealed by ASC06-IgG1

**Fig. 4:**
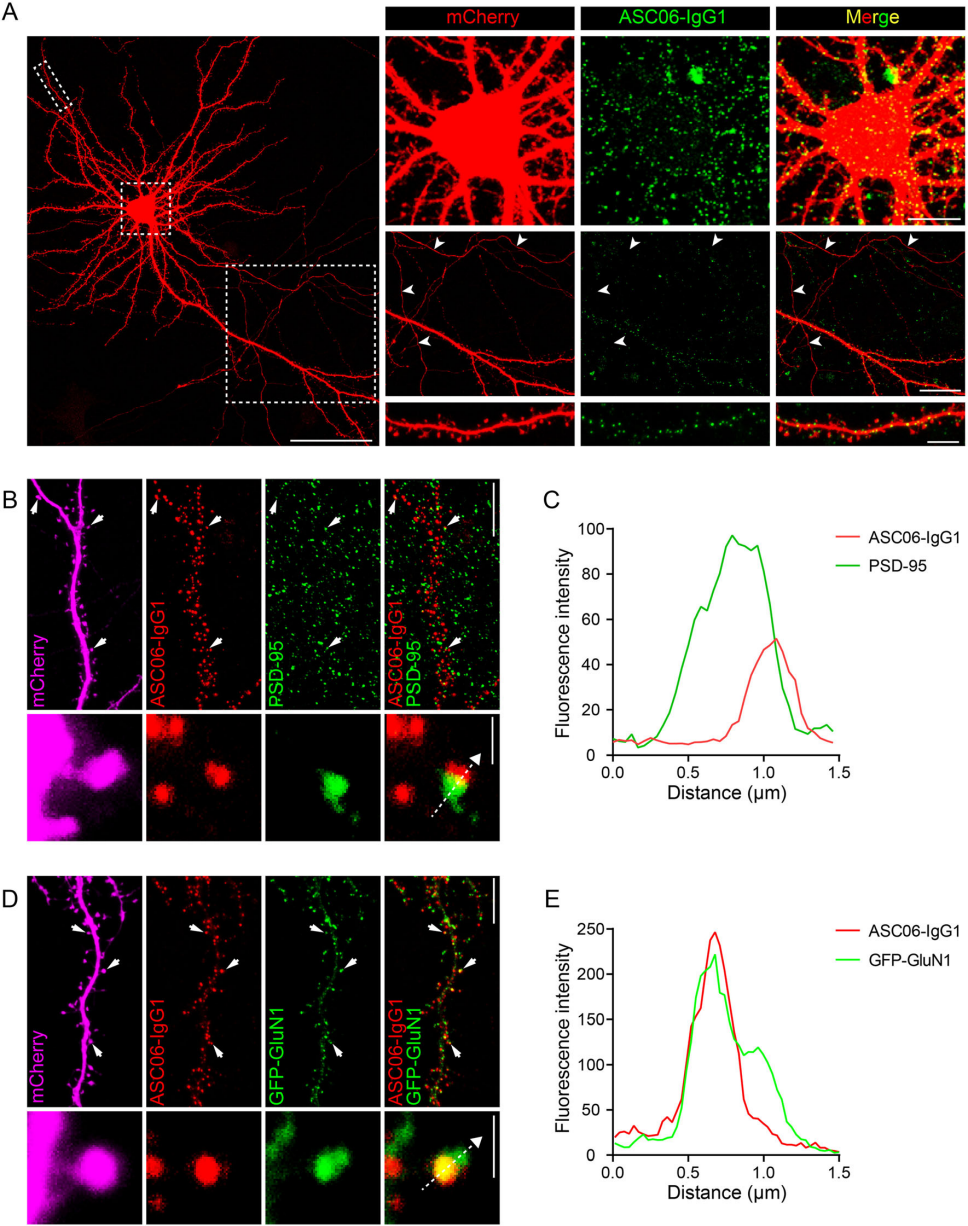
Plasma membrane distribution of neuronal hASIC1a labeled by the hASIC1a ectodomain antibody. **A** Representative images of neuronal surface hASIC1a stained by antibody ASC06-IgG1. Surface labeling by ASC06-IgG1 (green) and immunostaining for mCherry (red) in *Asic1a*^−/−^ neurons co-transfected with hASIC1a and mCherry. Boxed areas are enlarged to show the location of surface hASIC1a in soma, axon and dendrite. Arrowheads indicate axons; scale bars: left, 50 μm; top right, 10 μm; middle right, 20 μm; bottom right, 5 μm. **B**, **C** Relative localization of surface hASIC1a with PSD-95. **B** A representative dendrite segment and a magnified spine with surface staining by ASC06-IgG1 (red) and immunostaining for PSD-95 (green) and mCherry (magenta) in an *Asic1a*^−/−^ neuron co-transfected with mCherry and hASIC1a. Scale bars: upper right, 10 μm; lower right, 1 μm. **C** Plot for the line profile of ASC06-IgG1 (red) and PSD-95 (green) along the dashed lines in the spine shown in (**B**). **D**, **E** Relative localization of surface hASIC1a with GluN1. **D** A representative dendrite segment and a magnified spine with surface staining by ASC06-IgG1 (red) and immunostaining for GFP-GluN1 (green) and mCherry (magenta) in an *Asic1a*^−/−^ neuron co-transfected with hASIC1a, GFP-GluN1 and mCherry. Scale bars: upper right, 10 μm; lower right, 1 μm. **E** Plot for the line profile of ASC06-IgG1 (red) and GFP-GluN1 (green) along the dashed line in the spine shown in (**D**).

Given that ASC06-IgG1 labeled hASIC1a much better than rodent ASIC1a at the cell surface (Fig. 3B, C and F), we focused on the surface localization of WT hASIC1a heterologously expressed in *Asic1a*^−/−^ mouse neurons. Following surface labeling with ASC06-IgG1 under non-permeabilized conditions, surface hASIC1a was found to cluster across the soma surface and along the dendritic membrane, with no detection in axons (Fig. 4A). Consistent with surface labeling by extracellularly-tagged hASIC1a (Fig. 2A), surface-exposed hASIC1a was found to cluster not only on dendritic spines, but also on shafts (Fig. 4A). These results confirm the preferential somatodendritic localization of surface hASIC1a.

With additional immunostaining for PSD-95 (Fig. 4B and C) or GFP-GluN1 (Fig. 4D and E), the surface hASIC1a puncta labeled by ASC06-IgG1 also appeared to be either closely adjacent to (Fig. 4B and C) or co-localized with (Fig. 4D and E) these excitatory synaptic markers in spine heads. These results are entirely consistent with that obtained using the ex-tagged hASIC1a construct and they strengthen the conclusion of postsynaptic targeting of surface hASIC1a. Therefore, we not only obtained another powerful tool to label WT hASIC1a on the PM, but also corroborated the neuronal and subcellular localization of function-relevant surface hASIC1a in cortical neurons.

### Visualizing Membrane Trafficking of hASIC1a in Live Neurons

**Fig. 5:**
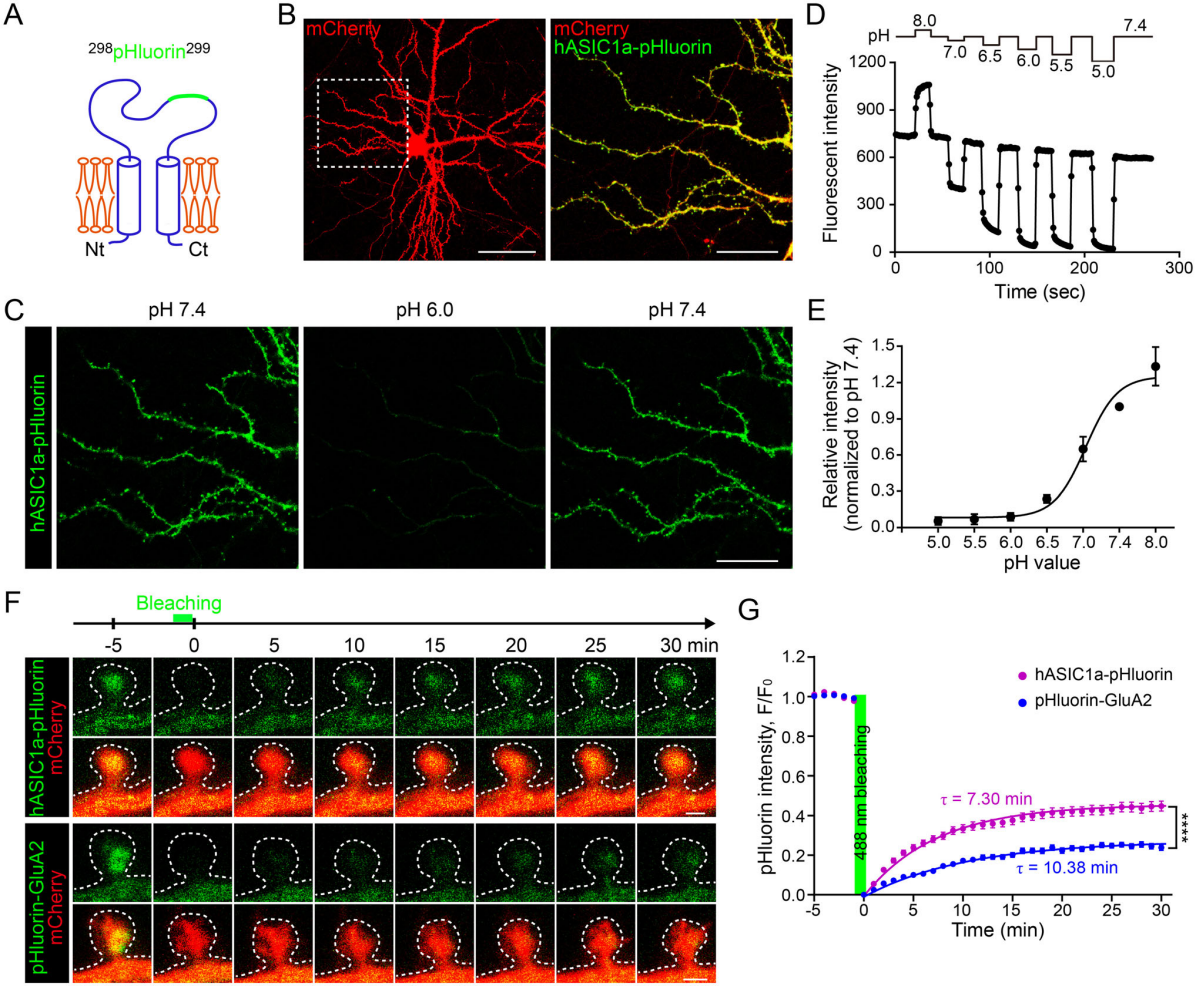
Membrane trafficking of hASIC1a in dendritic spines of live neurons. **A** Diagram of ex-tagged hASIC1a-^298^pHluorin^299^; pHluorin is inserted between Asp^298^ and Leu^299^ of the hASIC1a extracellular loop. **B** Representative images of the expression and distribution of hASIC1a-pHluorin in *Asic1a*^−/−^ neurons. hASIC1a-pHluorin and mCherry were co-transfected into ASIC1a-null neurons and images were captured under live condition at DIV 18. Scale bars: left panel, 50 μm; right panel, 20 μm. **C** Representative images showing the pH sensitivity of hASIC1a-^298^pHluorin^299^ fluorescence. *Asic1a*^−/−^ neurons transfected with hASIC1a-^298^pHluorin^299^ were perfused back and forth with solutions of pH 7.4 and 6.0 as indicated. Scale bar: 20 μm. **D** hASIC1a-^298^pHluorin^299^ fluorescence intensity in extracellular solutions (ECS) of pH 8.0, 7.4, 7.0, 6.5, 6.0, 5.5, and 5.0 as indicated. **E** pH dose-response curve of hASIC1a-^298^pHluorin^299^ fluorescence. Data points represent the mean ± SEM of *n =* 9 neurons and are fitted with the Hill equation to yield an IC_50_ of pH 5.08. **F**, **G** Membrane trafficking of pHluorin-GluA2 and hASIC1a-^298^pHluorin^299^ in excitatory synapses. **F** Representative time-lapse images of FRAP in dendritic spines of WT rat neurons transfected with mCherry plus pHluorin-GluA2 or hASIC1a-^298^pHluorin^299^. After 5 min of baseline recording, the spine head is bleached with the 488 nm laser for 1 min, followed by another 30 min of recording. Scale bars: 0.5 μm. **G** Quantification of pHluorin fluorescence recovery. At 30 min, the fluorescence returns to 44.87 ± 2.53% (*n =* 54 spines) of baseline for hASIC1a-^298^pHluorin^299^ and 23.85 ± 1.40% (*n =* 49 spines) for pHluorin-GluA2. Data points represent the mean ± SEM, and those after bleaching are fitted with an exponential function which yields the solid lines and τ_pHluorin-GluA2_ = 10.38 min and τ_hASIC1a-pHluorin_ *=* 7.30 min. ****P < 0.0001, two-way ANOVA multiple comparisons.

The data above describe surface hASIC1a localization on static images of fixed samples. To gain information about the mobility of hASIC1a on the PM of live neurons, we replaced the extracellular HA tag with the pH-sensitive GFP variant pHluorin, which is commonly used as a probe for monitoring synaptic vesicle fusion [69], to create hASIC1a-^298^pHluorin^299^ (Fig. 5A). Before delivery to the PM, the fluorescence of hASIC1a-^298^pHluorin^299^ is quenched in the relatively acidic environment of intracellular vesicles. Delivery to the cell surface exposes the pHluorin to the neutral pH environment of the extracellular fluid and allows it to fluoresce. Upon expression in *Asic1a*^−/−^ neurons, hASIC1a-^298^pHluorin^299^ also targeted to dendrites, including spines (Fig. 5B). Live cell imaging showed pH-dependent changes in pHluorin fluorescence in response to the perfusion of extracellular solutions with different pH values (Fig. 5C). Specifically, the fluorescence intensity decreased by >95% at pH <6.0, indicating that the residual fluorescence from hASIC1a-^298^pHluorin^299^ present in cytoplasmic compartments accounted for very little of the overall signal detected under our experimental conditions. Quantification of the fluorescence intensity revealed that the pHluorin signal at pH 7.4 was relatively stable during multiple rounds of extracellular pH changes, with a <20% decrease over a period of 4 min, and the probe was most sensitive to pH changes between pH 8.0 and pH 6.5 (Fig. 5D and E, and Video S1). These observations demonstrate that the inserted pHluorin is readily available on the PM to detect extracellular pH changes in live cells and able to indicate surface hASIC1a under live conditions.

We then used hASIC1a-^298^pHluorin^299^ to examine the dynamics of synaptic hASIC1a in live neurons and compared it with that of pHluorin-GluA2, which represents constitutive trafficking of AMPA receptors [70–73]. After co-expressing hASIC1a-pHluorin or pHluorin-GluA2 with mCherry into WT rat cortical neurons, we performed FRAP experiments on DIV 18–21. Following baseline recording, the fluorescence in the head of a mushroom spine was photo-bleached using the 488 nm laser with the Nikon A1R confocal microscope for 1 min, which reduced the pHluorin signal to near the background level (Fig. 5F). The recovery of the signal was recorded for an additional 30 min. Interestingly, while the fluorescence of pHluorin-GluA2 returned to only ~24% of baseline at 30 min after photobleaching, that of hASIC1a-pHluorin recovered to ~45% of baseline (Fig. 5G). The rate of recovery, based on the time constant, τ, was also markedly faster for hASIC1a-pHluorin (τ = 7.30 min) than pHluorin-GluA2 (τ = 10.38 min) (Fig. 5G). We also conducted FRAP by bleaching dendritic segments and saw that hASIC1a-pHluorin fluorescence still recovered faster than pHluorin-GluA2 (Fig. S2A and B). These observations suggest that hASIC1a is more mobile than AMPA GluA2 receptors under basal conditions both in synaptic and non-synaptic regions of cortical dendrites.

### Lateral Movement of Surface hASIC1a on the Plasma Membrane in Live Neurons

**Fig. 6:**
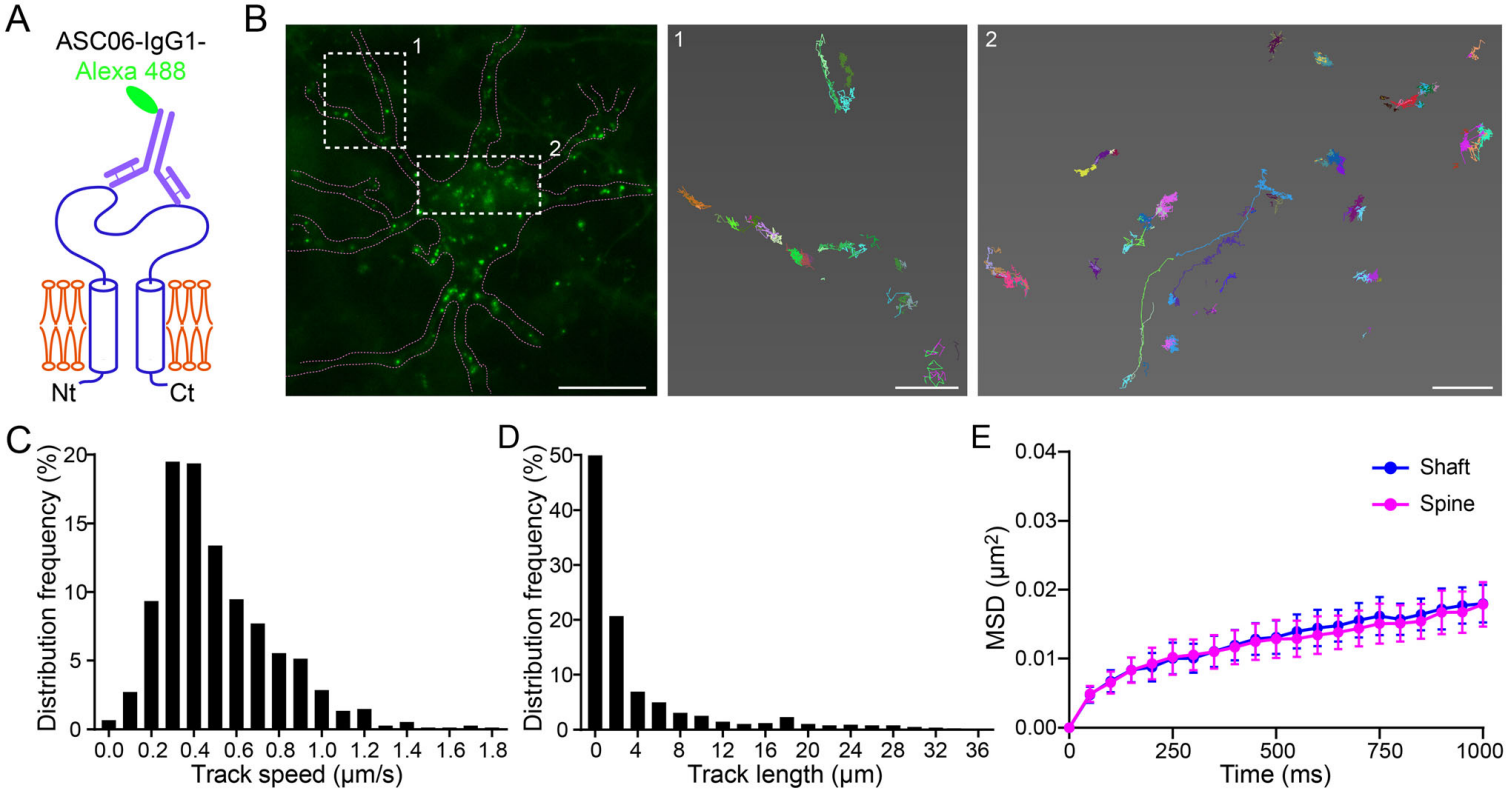
Lateral mobility of neuronal surface hASIC1a. **A** Diagram of direct labeling of surface hASIC1a by Alexa 488-conjugated ASC06-IgG1. **B**–**D** SPT experiments on neuronal surface hASIC1a. *Asic1a*^−/−^ neurons transfected with hASIC1a were incubated with a low concentration of ASC06-IgG1-Alexa 488. SPT was performed for 1 min–2 min using TIRF microscope at 20 Hz. **B** Representative image of a neuron labeled by ASC06-IgG1-Alexa 488 under live conditions (left panel) and enlarged reconstructed trajectories of surface hASIC1a clusters in the boxed areas (middle and right panels). Scale bars: left panel, 10 μm; middle and right panels, 1 μm. **C**, **D** Quantification of SPT on distribution frequencies of track speeds (**C**) and track lengths (**D**) of surface hASIC1a particles (*n =* 681 particles counted). Track speed = track length/track time. **E** Mean square displacement (MSD) as a function of time for surface hASIC1a clusters on dendritic shafts and spines. Data points represent the mean ± SEM of 17 cells. Paired *t* test on area under the curve showed no significant difference between shafts and spines.

Different forms of trafficking pathways permit the rapid supply of transmembrane neurotransmitter receptors, including exocytosis from intracellular reservoirs to the PM and lateral movement on the cell surface [74]. Single-particle tracking (SPT) techniques have been applied to investigate the lateral mobility of single molecules of AMPA [63, 75, 76] and GABA receptors [62, 77], which tremendously helps to reveal activity-dependent ion channel mobility on neuronal membranes at high spatiotemporal resolution. To test if the hASIC1a ectodomain antibody can be used for SPT, we labeled live *Asic1a*^−/−^ cortical neurons expressing WT hASIC1a with Alexa 488-conjugated ASC06-IgG1 (Fig. 6A) and then acquired high-frequency images at 20 Hz for 1 min–2 min using a TIRF microscope (Fig. 6B and Video S2). Trajectories were then constructed for Alexa 488-labeled particles. We focused on well-separated particles along the dendrites with low fluorescence intensities, which likely represented single or minimal antibody labeling (Fig. S3). Nearly all surface hASIC1a particles exhibited lateral movement (Fig. 6C and Video S2). While a majority of the particles displayed local mobilization, some showed long-range transport (Fig. 6D and Video S2), indicating diverse patterns of lateral mobility of surface hASIC1a on the dendritic PM. As previously described [62–64], we used MSD, representing the surface explored by the channels per unit time, to evaluate the lateral mobility of surface ASICs containing hASIC1a. The results showed that most of the fluorescent puncta were spatially confined within ~0.02 μm^2^ and there was no significant difference in the surface hASIC1a mobility between dendritic shafts and spines (Fig. 6E). The diffusion coefficient was difficult to estimate with the current sampling frequency.

Moreover, although the number remained low for the labeling of cultured rat primary cortical neurons with ASC06-IgG1-Alexa 488, the endogenous rASIC1a (Fig. S4A) showed patterns of lateral mobility similar to the heterologously-expressed hASIC1a (Fig. 6B). The MSD plot of endogenous surface rASIC1a in dendritic shafts was similar to that in spines (Fig. S4B), in line with that of transfected hASIC1a (Fig. 6E). Therefore, the fluorophore-conjugated hASIC1a antibody holds promise for further investigations to monitor the activity-induced synaptic dynamics of hASIC1a at the single-particle level.

### BDNF Regulation of ASIC1a Surface Expression and Dynamic Trafficking in Cortical Neurons

**Fig. 7:**
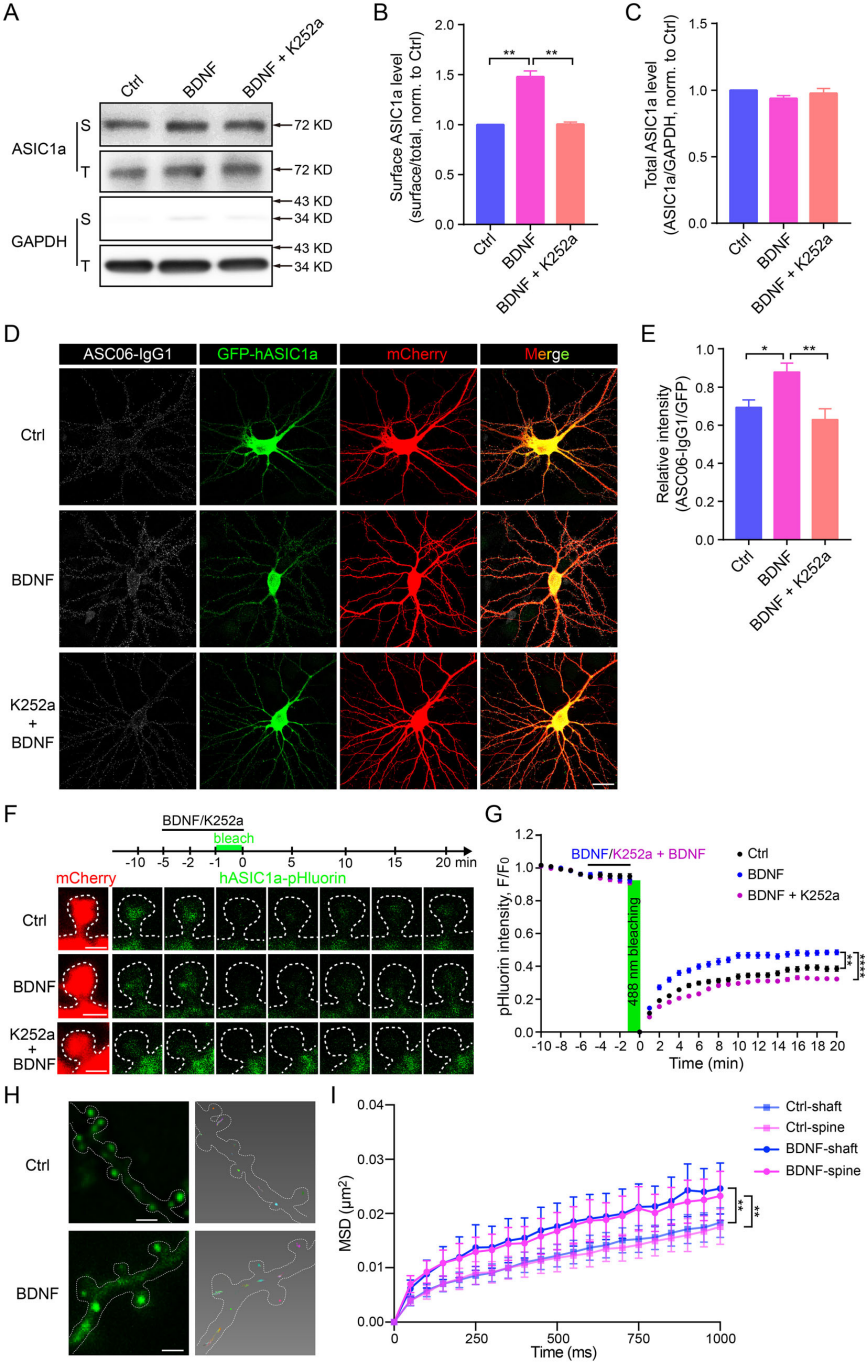
Enhanced spatiotemporal dynamics of neuronal surface ASIC1a underlying BDNF treatment. **A**–**C** Cultured rat cortical neurons treated with vehicle (Ctrl), BDNF (20 ng/mL) or BDNF plus the pan-Trk inhibitor, K252a (200 nmol/L) for 5 min before surface biotinylation. **A** Representative blots of surface (biotinylated, S) and total (T) ASIC1a, with GAPDH as a cytoplasmic protein control. **B** Quantification of surface ASIC1a levels by S/T ratio (normalized to Ctrl). Data are presented as the mean ± SEM. [BDNF/Ctrl, 1.48 ± 0.02; BDNF/(K252a + BDNF), 1.47 ± 0.01; (K252a + BDNF)/Ctrl, 1.00 ± 0.02, **P < 0.01, *n =* 4 experiments, no significant difference between Ctrl and BDNF + K252a, one-way ANOVA multiple comparison]. **C** Quantification of total ASIC1a levels by ASIC1a/GAPDH ratio (normalized to Ctrl). Data are presented as the mean ± SEM. [BDNF/Ctrl, 0.94 ± 0.01; BDNF/(K252a + BDNF), 0.96 ± 0.04; (K252a + BDNF)/Ctrl, 0.98 ± 0.02, *n =* 4 experiments, no significant difference between any two groups, one-way ANOVA multiple comparison. **D**, **E** *Asic1a*^−/−^ neurons co-transfected with GFP-hASIC1a and mCherry were treated with vehicle, BDNF, or BDNF plus K252a as above before surface staining. **D** Representative images of surface hASIC1a stained by ASC06-IgG1, as well as that of total hASIC1a by GFP and neuron morphology by mCherry (scale bar, 20 μm). **E** Quantification of surface hASIC1a level based on ASC06-IgG1/GFP intensity ratio. Data are presented as the mean ± SEM. [Vehicle, 0.69 ± 0.05 (*n =* 11 neurons); BDNF, 0.88 ± 0.05 (*n =* 10 neurons); BDNF + K252a, 0.63 ± 0.06 (*n =* 8 neurons), *P < 0.05, **P < 0.01, no significant difference between Ctrl and BDNF + K252a, one-way ANOVA multiple comparison. **F**, **G** FRAP assays of dendritic spines of *Asic1a*^−/−^ neurons co-transfected with mCherry and hASIC1a-^298^pHluorin^299^. After 5 min of baseline recording, neurons were perfused with ECS containing vehicle, BDNF, or BDNF plus K252a for another 5 min. Then a selected mushroom spine head was bleached with the 488 nm laser for 1 min, followed by another 20 min of recording. **F** Representative time-lapse images (scale bars, 1 μm). **G** Quantification of FRAP. After 20 min of recovery, the pHluorin fluorescence returns to 40.20 ± 1.66% (*n =* 122 spines) of baseline for Ctrl, 49.98 ± 1.48% (*n =* 100 spines) for BDNF, and 32.22 ± 1.05% (*n =* 134 spines) for K252a + BDNF. Data points represent the mean ± SEM, **P < 0.01, ****P < 0.0001, no significant difference between Ctrl and BDNF + K252a, two-way ANOVA multiple comparison. **H**, **I** Effect of BDNF on lateral mobility of surface hASIC1a in dendritic shafts and spines. **H** Representative images and reconstructed trajectories of surface hASIC1a SPT (scale bars, 1 μm). **I** MSD *versus* time plot for the mobility of surface hASIC1a puncta on dendritic shafts and spines with or without BDNF treatment. Data points represent the mean ± SEM, *n =* 19 cells for Ctrl and *n =* 21 cells for BDNF-treated. Paired *t* test on area under the curve showed no significant difference between shafts and spines in either the Ctrl or BDNF treatment group, but **P < 0.01 for Ctrl *versus* BDNF-treated at either shafts or spines.

In order to test the newly-developed tools and explore the mechanisms underlying synaptic activity regarding ASIC1a, we introduced a stimulus paradigm to assess the dynamic trafficking of hASIC1a. BDNF regulates an activity-dependent form of synaptic plasticity [46–54] and neuronal growth and differentiation [40–45]. Previously, we used a central pain-sensitization model to show that BDNF upregulates the surface expression of ASIC1a in spinal dorsal horn neurons [57]. Since ASIC1a function has been implicated in synaptic plasticity [21, 25, 27, 78], we asked whether membrane trafficking of ASIC1a in the dendritic and synaptic surface of cortical neurons responds to strengthening of neuronal or synaptic activity by BDNF. Here, we first showed that BDNF treatment increased the surface expression of endogenous ASIC1a in cultured rat cortical neurons, as revealed by surface biotinylation (Fig. 7A and B), without affecting the total ASIC1a level (Fig. 7A and C). The pan-Trk inhibitor K252a abolished this effect of BDNF (Fig. 7A and B). Second, in *Asic1a*^−/−^ mouse cortical neurons transfected with GFP-hASIC1a, BDNF also increased the surface-expressed hASIC1a, as detected by immunostaining with ASC06-IgG1 under non-permeabilized conditions, and the effect was abolished by including K252a during the BDNF treatment (Fig. 7D and E). These results confirmed the ability of BDNF to induce membrane surface expression of ASIC1a in cortical neurons.

Next, to test whether and how BDNF regulates synaptic ASIC1a mobility, we treated *Asic1a*^−/−^ cortical neurons expressing hASIC1a-pHluorin with BDNF or BDNF plus K252a for 5 min and applied photobleaching during the last minute of treatment. The FRAP measurements showed that, at 20 min after photobleaching, hASIC1a-pHluorin fluorescence in the mushroom spine heads recovered to nearly 50% of baseline in the BDNF-treated neurons, which was markedly faster and more complete than those in the vehicle-treated (PBS) control neurons and neurons treated with BDNF plus K252a, showing restoration to 40.2% and 32.2%, respectively (Fig. 7F and G). Moreover, BDNF clearly accelerated the lateral mobility of surface hASIC1a (Fig. 7H and I) and significantly increased the MSD by ~30% on both the shafts and spines (Fig. 7I) in *Asic1a*^−/−^ cortical neurons transfected with hASIC1a, demonstrating the stimulatory effect of BDNF on the lateral movement of dendritic surface hASIC1a. Together, these data demonstrated the BDNF regulation of hASIC1a membrane trafficking, including both forward trafficking and lateral mobility, in cortical neuron dendrites and spines, consistent with the involvement of hASIC1a dynamics in synaptic activity and excitatory synaptic function.

## Discussion

### Labeling and Visualizing Function-Relevant hASIC1a on the Neuronal Plasma Membrane

As major proton receptors [19–21, 79, 80], ASICs have multiple functions in nervous systems [6–9]. Lacking effective labeling tools for endogenous ASIC1a, especially for cell-surface channels, has largely hampered the exploration of mechanisms by which H^+^/ASIC1a is involved in biological processes. To address these issues, probes that label the extracellular regions of ASIC1a are of utmost importance. However, not only is it challenging to develop specific antibodies for immunostaining endogenous ASIC1a in neurons [21, 23, 36, 81], but even for studies that revealed the subcellular localization of N-terminus-tagged ASIC1a following heterologous expression in brain slices, the probe was added at the cytoplasmic side [23], leaving the question of the neuronal surface distribution of ASIC1a unanswered. Unlike ionotropic glutamate receptors, of which the surface location and regulation have been intensively studied by antibodies recognizing the free extracellular N-terminus that is amenable to antibody preparation, recognition and surface labeling [61, 63, 76], such an approach was not readily available for ASIC1a due to the lack of suitable antibodies that recognize the flexible extracellular loop.

Attempts have been made to examine surface ASIC1a with an HA epitope inserted into its extracellular loop [57, 58, 66]. Except for revealing surface ASIC1a in *Xenopus* oocytes or cell lines or in neuronal branches near the soma under conditions of extracellular staining, the labeling efficiency of the HA-tagged ASIC1a was unsatisfactory, especially for highly-specialized subcellular structures such as dendritic spines. Another issue is that most studies have been performed in rodents or with rodent ASIC1a and relatively little is known about the function and regulation of hASIC1a. Here, we applied two different approaches to label and visualize surface hASIC1a in cortical neurons (Table 1). First, we redesigned the strategy of inserting the HA tag into the hASIC1a ectodomain for surface labeling and identified hASIC1a-^298^HA^299^ to be able to efficiently indicate surface-exposed channels in both dendritic shafts and spines, in addition to the soma and primary branches, with minimal influence on channel localization, trafficking, and function. Further engineering with this insertion site using pHluorin allowed the examination of hASIC1a dynamics on the surface of excitatory synapses under live conditions. Second, we used the recently described hASIC1a ectodomain antibody, ASC06-IgG1 [59], for surface channel labeling, and by conjugation with Alexa 488, we also created a functional probe that permits monitoring lateral movement of the surface-localized hASIC1a in the dendrites of live cortical neurons *via* SPT. Although there may be still some drawbacks for the current version of the toolbox, it indeed provides the field with multiple available solutions for labeling and visualizing surface hASIC1a.

**Table 1:** Toolbox for labeling and visualization of cell-surface hASIC1a

	Surface labeling (ICC/IF)	Dynamic trafficking (live cell imaging)	Endogenous labeling (ICC/IF)	Channel function
Tag_N-terminus_-hASIC1a	No	No	No	Normal
hASIC1a-^298^HA^299^	Yes	/	No	Reduced
hASIC1a-^298^pHluorin^299^	/	Yes, FRAP	/	Reduced
ASC06-IgG1	Yes	/	Yes	/
ASC06-IgG1-Alexa 488	/	Yes, TIRFM/SPT	Yes	/

hASIC1a-^298^HA^299^ and ASC06-IgG1 can be used to label surface hASIC1a in heterologously-expressed neurons. hASIC1a-^298^pHluorin^299^ is suitable for visualizing general membrane trafficking of ASIC1a in transfected neurons. Fluorophore-conjugated ASC06-IgG1 is able to track the lateral movement of surface hASIC1a at the single-particle level. ASC06-IgG1 and its modified products are also suitable for labeling endogenous hASIC1a in human-derived neurons and rat brain neurons. N-terminus-tagging does not impair the acid-activated current of hASIC1a, while extracellular tagging affects the channel function to some extent. ICC, immunocytochemistry; IF, immunofluorescence. FRAP, fluorescence recovery after photobleaching; TIRFM, total internal reflection fluorescent microscopy; SPT, single-particle tracking.

### Synaptic Targeting and Spatiotemporal Dynamics of Surface hASIC1a in Cortical Neurons

With the toolbox, we resorted to the heterologous expression of hASIC1a in primary cortical neurons from *Asic1a*^−/−^ mice to examine the surface distribution of hASIC1a in dendrites. The ASIC1a-null background helped to evade any potential impact of preexisting mASIC1a on the surface localization of the heterologously-expressed hASIC1a. Using the optimized antibody-feeding strategy in cortical neurons, we showed that surface hASIC1a is clustered preferentially on the PM of somata and dendrites, and hardly detectable on axons, similar to the report on total ASIC1a distribution [23]. Surface hASIC1a was enriched in dendritic spines, clustering in and around post-synaptic membranes, where excitatory synaptic transmission occurs. There was also abundant surface hASIC1a on dendritic shafts, which may serve not only as a sensor of extra-synaptic or global environmental pH changes but also as a pool for updating or exchange of functional channels for synaptic activity through lateral diffusion. Importantly, the staining of WT hASIC1a with ASC06-IgG1 yielded results similar to staining hASIC1a-^298^HA^299^ with the anti-HA antibody, validating the observations made with either approach. These results support the idea that ASIC1a plays its modulatory role in synaptic function mainly in post- rather than pre-synaptic regions, consistent with the notion that synaptically-located ASIC1a acts as a proton receptor to sense pH transients concomitant with transmitter release [23, 24, 79, 82, 83].

By imaging live cortical neurons with hASIC1a-pHluorin and ASC06-IgG1-Alexa 488, we showed the synaptic hASIC1a to be highly dynamic. In FRAP experiments, the fluorescence recovery of hASIC1a-^298^pHluorin^299^ in dendritic spines, reflecting the exchange between synaptic membrane and cytosol, or between synaptic and extra-synaptic membrane, appeared to be faster and more complete than pHluorin-GluA2. This implies more active trafficking of the functional hASIC1a than GluA2 at the postsynaptic membrane, although differences in internal membrane structures and pH environments associated with these two channel types may also contribute to the difference in the FRAP experiments. Using ASC06-IgG1-Alexa 488 for SPT, we found that most of the surface hASIC1a, as well as the endogenous rASIC1a, on dendritic shafts and spines displayed lateral movement on the PM. While some of the labeled particles showed high mobility and long-distance migration, more of them exhibited relatively low speed and local mobility. Of note, ASIC1a can form both homotrimeric channels themselves and heterotrimers with ASIC2a or ASIC2b in the rodent brain, amongst which ASIC1a is the key determinant of acid-evoked current. Our study for the first time revealed the spatiotemporal dynamics of surface ASICs containing ASIC1a in excitatory synapses of live neurons, demonstrating that membrane trafficking of function-relevant ASIC1a at the postsynaptic surface is highly active, which may be intimately linked to the modulation of synaptic strength.

Although the novel antibody ASC06-IgG1 brought hope to the examination of endogenous surface channels, the neuronal distribution of endogenous surface hASIC1a remains to be determined. Human neurons or resected tissue [84] from brain tumor patients or epileptic patients may be used to address this question in the future. Alternatively, neurons derived from human neural stem cells may also provide the opportunity to resolve the cell-type specific distribution of endogenous hASIC1a. However, an obvious caveat is that none of these preparations is considered to completely represent native neurons. Moreover, to obtain more detailed information about the precise localization of hASIC1a in synapses, including both excitatory and inhibitory synapses, super-resolution microscopy is needed in future studies.

### Dynamic Trafficking of Neuronal Surface hASIC1a and Synaptic Function

Previous studies have demonstrated the importance of ASIC1a trafficking in neurological disorders. For instance, the constitutive endocytosis of ASIC1a protects neurons from acidosis-induced cell death [66] and BDNF regulation of surface ASIC1a expression contributes to the central sensitization of pain [57]. Physiologically, BDNF is a well-established modulator of both synaptic plasticity [46–54] and neural development [40–45]. Thus, we attempted to determine the physiological relevance of hASIC1a membrane trafficking following BDNF treatment. We used surface-expressed hASIC1a as the probe to monitor the dynamics of function-relevant channels on dendritic membranes and confirmed that BDNF upregulated the surface expression of ASIC1a in cortical neurons. Moreover, like the AMPA receptors, for which multiple studies have used pHluorin- or SEP-tagged constructs to demonstrate the fundamental role of membrane trafficking during synaptic plasticity *in vitro* and *in vivo* [85, 86], the membrane dynamics of hASIC1a-^298^pHluorin^299^ expressed in cultured *Asic1a*^−/−^ mouse cortical neurons were also enhanced by BDNF.

Although the activity-dependent transport of ASIC1a-GFP in neurons has been described [57], it does not inform membrane trafficking of functional surface ASIC1a in synapses. In addition to forward trafficking, a growing body of evidence also suggests that synaptic activity requires the lateral mobility of AMPA receptors on post-synaptic membrane [63, 75, 76]. We found the lateral mobility of surface hASIC1a on both dendritic shafts and dendritic spines to be accelerated in response to BDNF stimulation. Therefore, BDNF appears to exert a generalized role in regulating ASIC1a trafficking in cortical neurons by promoting both its forward trafficking and lateral mobility. Accumulating evidence strongly suggests the crucial roles of ASIC1a in synaptic plasticity and associative learning and memory [21, 26, 80]. Acting as a proton receptor to sense the synaptic cleft acidification associated with neurotransmitter release, postsynaptic ASIC1a has been postulated to accomplish these roles by enhancing the depolarization of post-synaptic membrane and consequently affecting the strength of synaptic transmission [7, 8, 24, 83]. Yet, it remained unclear how ASIC1a is involved in synaptic activity. Our findings imply the involvement of hASIC1a membrane trafficking in neuronal/synaptic activity, and this may underlie the mechanism by which ASIC1a regulates synaptic plasticity.

In summary, we developed a new toolbox set to label and visualize functional cell-surface hASIC1a in cortical neurons, with emphasis on the correlation of hASIC1a trafficking and synaptic function. Using extracellular epitope tags and a novel antibody recognizing the hASIC1a ectodomain, we demonstrated the postsynaptic membrane targeting and dynamic trafficking of surface hASIC1a clusters in cortical neurons. BDNF-induced enhancement of synaptic hASIC1a trafficking provides a new perspective to couple synaptic strength with the spatiotemporal dynamics of hASIC1a. Equipped with these tools, future studies may use human-derived neurons, genetic engineering, super-resolution microscopy, and two-photon imaging *in vivo* to determine the nanoscale synaptic locations of endogenous hASIC1a, directly monitor behavior-related hASIC1a dynamics in animals, and consequently elucidate the mechanisms underlying H^+^ and ASIC1a-mediated synaptic plasticity, learning, and memory.

## Electronic supplementary material

Below is the link to the electronic supplementary material.

Supplementary material 1 (PDF 1252 kb)

Supplementary material 2 (PDF 9 kb)

Supplementary material 3 (MOV 9666 kb)

Supplementary material 4 (MOV 7277 kb)
