# Supplementary material for: Postsynaptic Targeting and Mobility of Membrane Surface-Localized hASIC1a
Source: Neurosci Bull. 2020 Sep 30;37(2):145–65. doi: 10.1007/s12264-020-00581-9 (PMC7870742; doi:10.1007/s12264-020-00581-9)
Supplement: Supplementary file 1 — Supplementary material 1 (PDF 1252 kb) [file 12264_2020_581_MOESM1_ESM.pdf]

Supplementary Figure and Legends

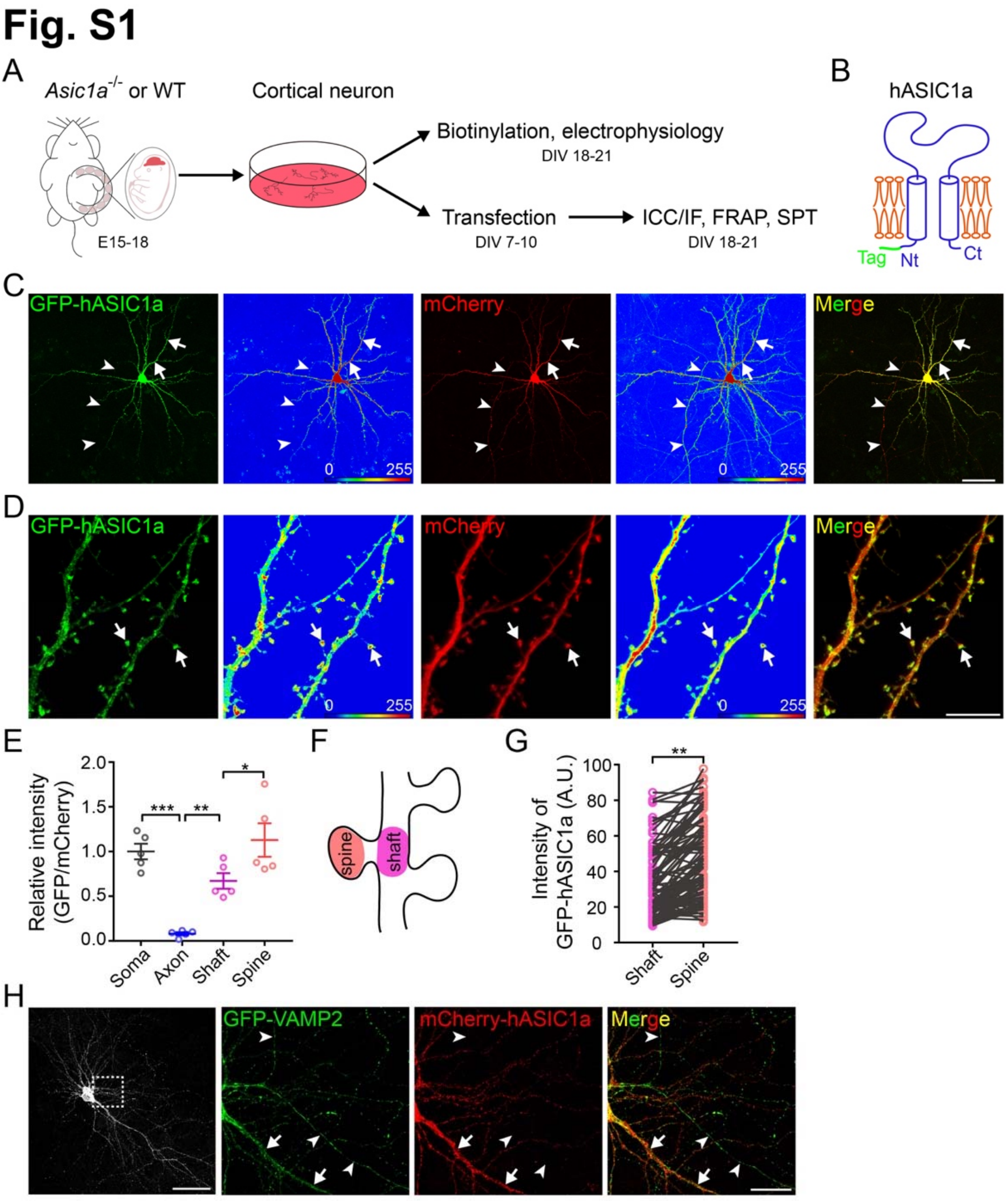

Fig. S1 Subcellular distribution of total hASIC1a in cortical neurons. A Schematic flow diagram for primary neuron experiments. Primary cortical neurons were isolated from fetal rats or fetal WT or *Asic1a*$^{-/-}$ mice. At DIV 7-10, neurons were transfected with the desired plasmids. At DIV 18-21, immunostaining, live cell imaging, or biochemical experiments were performed. ICC, immunocytochemistry; IF, immunofluorescence; FRAP, fluorescence recovery after photobleaching; SPT, single particle tracking. **B** Diagram of N-terminus-tagged hASIC1a (GFP/mCherry-hASIC1a). **C** Representative images of rat cortical neurons co-transfected with GFP-hASIC1a (green) and mCherry (red). Intensity graphs are shown right to the fluorescence images to reveal the abundance of GFP-hASIC1a and mCherry in different subcellular structures. Arrowheads indicate axon-like branches, while arrows indicate dendrites. Scale bar, 50 μm. **D** Representative images of dendritic segments showing GFP-hASIC1a and mCherry in dendritic spines (indicated by arrows) of *Asic1a*$^{-/-}$ mouse cortical neurons. Scale bar, 10 μm. **E** Quantification of GFP-hASIC1a distribution in neurons by fluorescence intensity ratio of GFP-hASIC1a/mCherry. Data represent mean ± SEM. The values are: soma, 1.00 ± 0.09; axon, 0.08 ± 0.02; dendritic shaft, 0.67 ± 0.09, and spine, 1.12 ± 0.19. $n = 5$ neurons. One-way ANOVA multiple comparison, $*P < 0.05$, $**P < 0.01$, $***P < 0.001$. **F, G** Quantification of the spine enrichment of GFP-hASIC1a by normalizing the spine signals to that of the shaft directly underneath the spine. Two-tailed paired Student's *t*-test, $**P < 0.01$, $n = 115$ spines. **H** Representative images of *Asic1a*$^{-/-}$ mouse cortical neurons co-transfected with axon vesicle marker GFP-VAMP2 and mCherry-hASIC1a. Arrowheads indicate GFP-VAMP2-positive axons, while arrows indicate dendrites. Scale bars: left panel, 50 μm; right panel, 20 μm.

Fig. S2

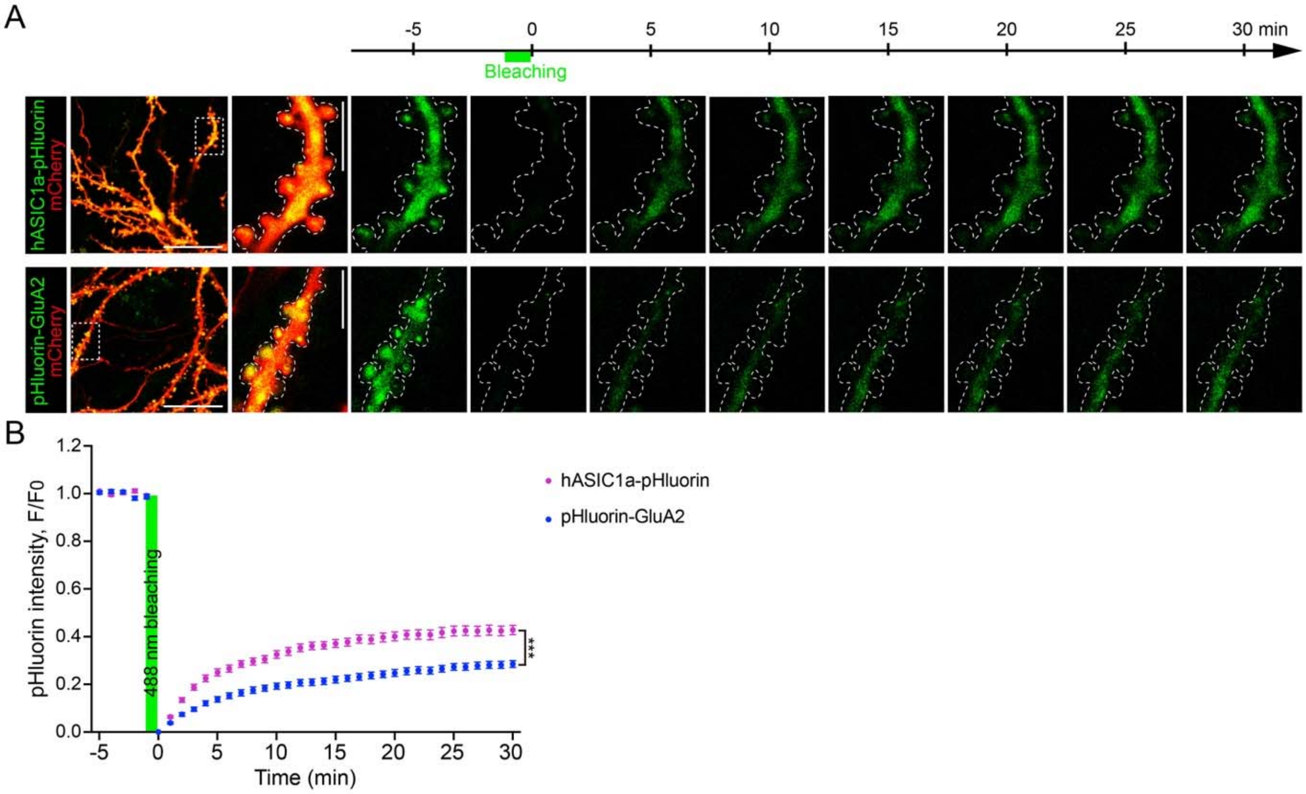

Fig. S2 Membrane trafficking of hASIC1a in the dendrites of live neurons. A Representative time-lapse images of FRAP in dendritic segments of WT rat neurons transfected with mCherry plus pHluorin-GluA2 or hASIC1a-^{298}pHluorin^{299}. After 5 min of recording the baseline, the spine head was bleached with the 488 nm laser for 1 min, followed by another 30 min of recording. Scale bars: left, 20 μm; enlarged, 5 μm. **B** Quantification of pHluorin fluorescence recovery. At 30 min, the fluorescence restored to 42.80% ± 1.92% (n = 47 dendritic fragments) of the baseline for hASIC1a-^{298}pHluorin^{299} and 28.56% ± 1.43% (n = 55 dendritic fragments) for pHluorin-GluA2. Data points represent mean ± SEM, Two-way ANOVA multiple comparisons, ***$P < 0.001$.

Fig. S3

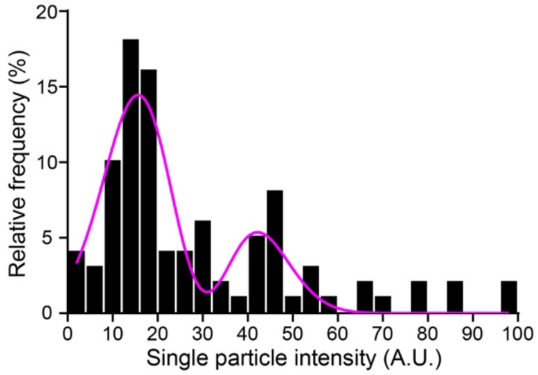

Fig. S3 Surface hASIC1a particle characterization in SPT experiment. Fluorescent intensity of individual surface hASIC1a particles labeled by ASC06-IgG1-Alexa 488 was analyzed and the representative frequency distribution histogram was based on Fig. 6B (95 particles). Particles within the major Gaussian curve (leftmost) are used for subsequent lateral mobility analysis (Fig. 6E, Fig. 7I and Fig. S4B).

Fig. S4

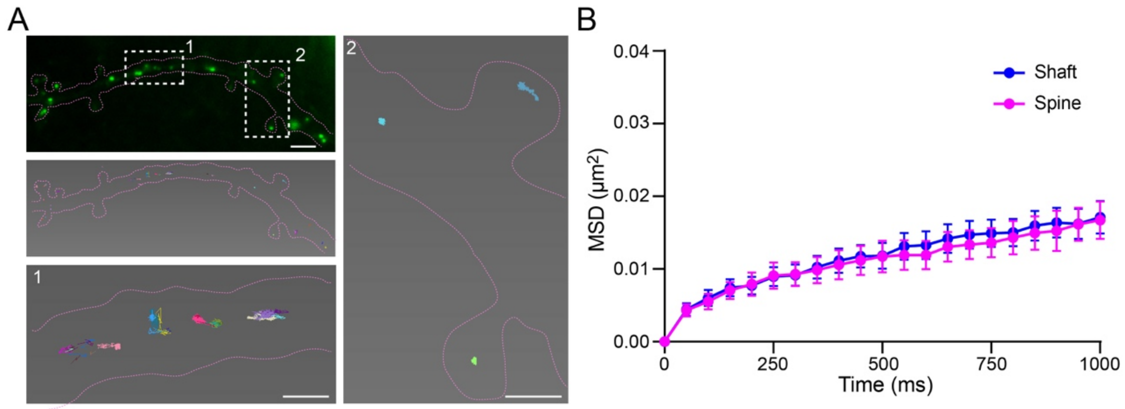

Fig. S4 Lateral mobility of endogenous surface rASIC1a in rat primary cortical neurons by SPT. **A** Representative image and trajectories of surface rASIC1a clusters on dendritic shafts and spines of rat cortical neurons. Scale bars: top left panel, 2 μm; bottom left and right panels, 1 μm. **B** MSD as a function of time for endogenous surface rASIC1a clusters on dendritic shafts and spines. Data points represent mean ± SEM, n = 19 cells. Paired t test on area under the curve showed no significant difference between shafts and spines.
