# Supplementary material for: Postsynaptic Targeting and Mobility of Membrane Surface-Localized hASIC1a
Source: Neurosci Bull. 2020 Sep 30;37(2):145–65. doi: 10.1007/s12264-020-00581-9 (PMC7870742; doi:10.1007/s12264-020-00581-9)
Supplement: Supplementary file 2 — Supplementary material 2 (PDF 9 kb) [file 12264_2020_581_MOESM2_ESM.pdf]

Supplementary Video Captions

Video S1. Changes of hASIC1a-pHluorin fluorescence in response to extracellular pH fluctuations. After transfection with hASIC1a-^{298}pHluorin^{299} and mCherry in *Asic1a*$^{-/-}$ neurons, live cell imaging with no delay showed pH-dependent changes in pHluorin fluorescence in response to the perfusion of extracellular solutions with different pH values.

Video S2. Single-particle tracking of surface hASIC1a by ASC06-IgG1-Alexa 488. *Asic1a*$^{-/-}$ neurons transfected with hASIC1a were incubated with ASC06-IgG1-Alexa 488 to label surface hASIC1a. Total internal reflection fluorescent microscope (TIRFM) was used to record lateral mobility of surface hASIC1a. Scanning was performed for 1 to 2 min at a frequency of 20-30 Hz.
